# Follicular Regulatory CD8 T Cells Impair the Germinal Center Response in SIV and *Ex Vivo* HIV Infection

**DOI:** 10.1371/journal.ppat.1005924

**Published:** 2016-10-07

**Authors:** Brodie Miles, Shannon M. Miller, Joy M. Folkvord, David N. Levy, Eva G. Rakasz, Pamela J. Skinner, Elizabeth Connick

**Affiliations:** 1 Division of Infectious Diseases, Anschutz Medical Campus, University of Colorado Denver, Colorado, United States of America; 2 Department of Immunology and Microbiology, School of Medicine, Anschutz Medical Campus, University of Colorado Denver, Colorado, United States of America; 3 Division of Infectious Diseases, University of Arizona, Arizona, United States of America; 4 Department of Basic Science, New York University College of Dentistry, New York, United States of America; 5 Wisconsin National Primate Research Center, University of Wisconsin-Madison, Wisconsin, United States of America; 6 Department of Veterinary and Biomedical Sciences, University of Minnesota, Minnesota, United States of America; Emory University, UNITED STATES

## Abstract

During chronic HIV infection, viral replication is concentrated in secondary lymphoid follicles. Cytotoxic CD8 T cells control HIV replication in extrafollicular regions, but not in the follicle. Here, we show CXCR5^hi^CD44^hi^CD8 T cells are a regulatory subset differing from conventional CD8 T cells, and constitute the majority of CD8 T cells in the follicle. This subset, CD8 follicular regulatory T cells (CD8 T_FR_), expand in chronic SIV infection, exhibit enhanced expression of Tim-3 and IL-10, and express less perforin compared to conventional CD8 T cells. CD8 T_FR_ modestly limit HIV replication in follicular helper T cells (T_FH_), impair T_FH_ IL-21 production via Tim-3, and inhibit IgG production by B cells during *ex vivo* HIV infection. CD8 T_FR_ induce T_FH_ apoptosis through HLA-E, but induce less apoptosis than conventional CD8 T cells. These data demonstrate that a unique regulatory CD8 population exists in follicles that impairs GC function in HIV infection.

## Author Summary

HIV is a chronic infection and is never completely cleared from the body, despite successful antiretroviral therapy that reduces plasma viral loads to undetectable levels and restores CD4 T cell counts. While undetectable in plasma, HIV is able to hide in various niches throughout the body. One such niche are CD4 T cells residing in the follicles and germinal centers of secondary lymphoid tissues. The dynamics of these regions that lead to persistence of HIV-infected cells remain unclear. However, recent evidence strongly suggests that CD8 cytotoxic T lymphocytes, which are able to kill HIV-infected cells outside of these regions, are present at low numbers in follicles and germinal centers. Here, we further advance these recent findings by showing that the few CD8 T cells within the follicle have potent regulatory functions rather than conventional cytotoxic functions. Thus, the CD8 T cells entering these regions of HIV persistence not only fail to kill HIV-infected cells, but promote impairments in humoral immunity. These findings identify a new obstacle that must be taken into account to improve immune responses and clearance of HIV.

## Introduction

In chronic HIV and SIV infection, viral replication is concentrated in B cell follicles in secondary lymphoid tissues [1–5], although factors that promote this are not fully understood. Follicular helper T cells (T_FH_), which reside in the secondary lymphoid follicles, are highly permissive to HIV [6] and exhibit anti-apoptotic properties [7, 8] which likely contributes to viral persistence. We have previously shown that virus-specific CD8 T cells are present at lower frequencies inside the follicle compared to outside the follicle in HIV and SIV infection [2, 9], which may contribute to impaired viral clearance in the follicle. While CD8 T cells are present in the follicle, little is known about the function of these cells. We have previously reported that CD4 follicular regulatory T cells (T_FR_) are increased in number, exhibit heighted regulatory capabilities, and impair T_FH_ proliferation and function in *ex vivo* HIV and *in vivo* SIV infection [7]. We hypothesized that follicular CD8 T cells may also have regulatory functions that further contribute to immune dysregulation in chronic HIV infection.

Regulatory CD4 T cell populations can be readily identified based on expression of CD25 [10, 11], and Foxp3 [12], their canonical transcription factor. Nevertheless, a consensus phenotype for CD8 Tregs has yet to be described. CD8 Tregs in the thymus and periphery of mice do not constitutively express Foxp3 [12], and Foxp3-expressing CD8 T cells do not encompass CD8 Treg populations [13]. CD8 Tregs have been described in humans, but have limited defining characteristics, and most lack Foxp3 [14]. Thus, it is essential to demonstrate regulatory function with any CD8 Treg phenotype [15, 16].

In mice, CD8 Treg function is dependent on B and T lymphocyte expression of Qa-1, the murine equivalent of HLA-E, which binds to the TCR of CD8 T cells [17–19]; CD8 Treg function correlates with the affinity and duration of this interaction [18, 20]. A specific subset of CXCR5^hi^CD44^hi^ CD8 Tregs (henceforth defined as CD8 T_FR_ in this work) was found to limit germinal center (GC) size and prevent autoimmune disease in mice [19]. The main targets of CD8 T_FR_ are CD4 T cells [17], specifically T_FH_ [19]. In autoimmune-prone mice, CD8 T_FR_ limit T_FH_ expansion and autoantibody production [21]. CD8 T_FR_ expressing CD122 (IL-2Rβ) in mice were also shown to inhibit CD8 T cell function through a mechanism involving IL-10 production, but not requiring TGFβ [22]. CD8 T_FR_ differ from conventional CD8 T cells in their potent suppressive mechanisms and their dependence on IL-15 for function [19]. Importantly, cells with the CD8 T_FR_ phenotype (CXCR5^hi^CD44^hi^ CD8+) have recently been identified in humans [23].

In the context of HIV infection there is limited evidence of CD8 Tregs. Stimulation of CD8 T cells isolated from HIV-infected patients with HIV peptides was shown to drive regulatory CD8 T cell function [24]. Suppressive function of HIV-specific CD8 T cells was further shown to be dependent on IL-10 production [25, 26]. These HIV-specific CD8 T cells that produced IL-10 lacked both CD25 and Foxp3, but were able to prevent HIV-specific cytolytic function [26]. Tim-3 has been previously shown to mediate CD4 Treg suppression [27] and impair virus-specific CD8 T cell responses [28]. CD8 T cells co-expressing PD-1 and Tim-3 have both exhausted effector function and produce high levels of IL-10 during chronic viral infection [29]. Upregulation of Tim-3 on CTL is strongly correlated with reduced cytotoxicity [30] and viral persistence [31]. Additionally, Tim-3+ CD8 T cells were increased in frequency and positively correlated with plasma viral load in progressive HIV infection [32]. Increased frequencies of CD8 T cells are also found in follicles of HIV-infected individuals compared to seronegative individuals [1], but little is known about their functional properties. No studies to date have evaluated CD8 T_FR_ in HIV or SIV infection and their potential role in HIV replication and persistence.

In this study, we describe CD8 T_FR_ phenotype and function in secondary lymphoid tissues during *ex vivo* HIV infection of human tonsils and *in vivo* SIV infection. We define CD8 T_FR_ as CD3+CD8+CXCR5^hi^CD44^hi^ cells and find that most follicular CD8 T cells in human and rhesus macaque secondary lymphoid tissues are CD8 T_FR_. CD8 T_FR_ exhibit an enhanced regulatory phenotype in the context of *ex vivo* HIV infection and chronic SIV infection and are expanded in chronic SIV infection. In contrast to conventional CD8 T cells, CD8 T_FR_ are able to impair T_FH_ effector function via Tim-3 and inhibit GC B cell IgG production. Further, CD8 T_FR_ modestly reduce HIV replication in T_FH_ and induce less T_FH_ apoptosis than conventional CD8 T cells in HIV infection *ex vivo*. Induction of T_FH_ apoptosis by CD8 T_FR_ is dependent on HLA-E. This work provides the first functional analysis of follicular CD8 T cell populations in the context of lentiviral infections and suggests that CD8 T_FR_ contribute to impaired T_FH_ function and GC dysfunction during HIV infection.

## Results

### Characterization of CD8 T_FR_ in ex vivo HIV-infected tonsils

**Fig 1:**
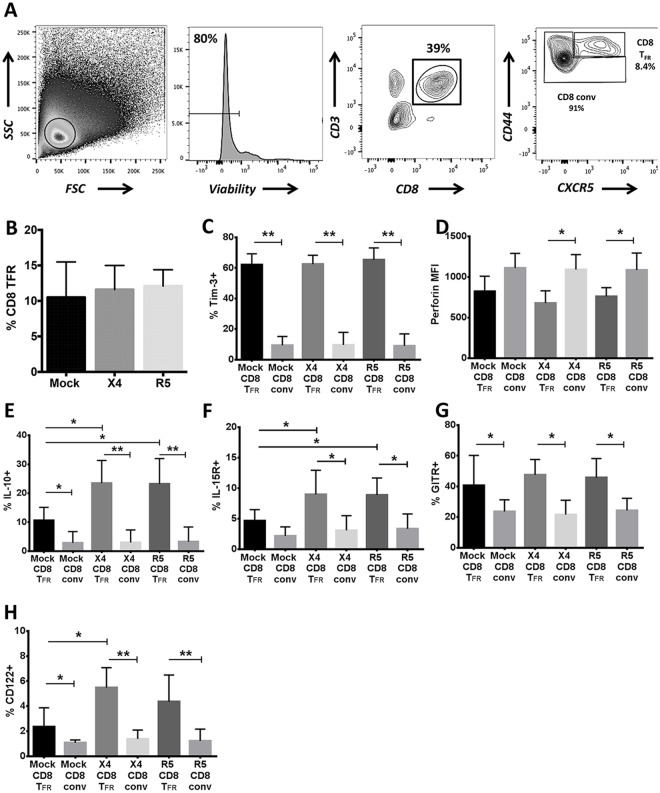
Human tonsil CD8 T_FR_ are distinct from conventional CD8 T cells. Disaggregated tonsil cell cultures were mock-spinoculated or spinoculated with X4- or R5-tropic HIV and cultured for 2 days (n = 8). (A) Flow gating strategy to determine viable, CD8 T_FR_ (CD3+CD8+CXCR5^hi^CD44^hi^) and all other CD3+CD8+ cells (CD8 conv). (B) The percentage of CD8 T_FR_ in mock- or HIV-spinoculated samples. The percent or MFI of CD8 T_FR_ and CD8 conv expressing (C) Tim-3, (D) perforin, (E) IL-10, (F) IL-15 receptor, (G) GITR, and (H) CD122 (IL-2Rβ). Graphs depict median and range. Statistical significance was determined by non-parametric one-way ANOVA (Friedman test) and is displayed as * = p<0.05 and ** = p<0.01.

First, we determined the fraction of follicular CD8 T cells within human tonsils that exhibit a CD8 T_FR_ phenotype two days following spinoculation with X4- or R5-tropic GFP reporter virus or mock spinoculation. CD8 T_FR_ are defined as viable CD3+CD8+CXCR5^hi^CD44^hi^ cells and conventional CD8 T cells (CD8 conv) defined as all other CD3+CD8+ cells as shown in representative flow cytometry plots in Fig 1A. The percentages of CD8 T_FR_ are not significantly altered 2 days after HIV spinoculation compared to mock-spinoculated cells (Fig 1B). Using flow cytometry counting beads to show the average of 3 experiments, we confirmed that CD8 T_FR_ did not increase numerically in the context of *ex vivo* HIV infection (S1 Fig). Additionally, we confirmed that percentages of CD8 T cells that are CD8 T_FR_ do not differ between day 0, prior to any spinoculation or cell culture, and day 2 following mock spinoculation and 2 days in culture media (S2 Fig).

Next, we analyzed CD8 regulatory and exhaustion molecules (Tim-3, CD122, IL-15R, and IL-10), the CD4 Treg mediator GITR [33], and perforin expression on CD8 T_FR_ and CD8 conv in the of context HIV infection *ex vivo*. First, there are no significant differences in CD122, IL-10 or perforin expression in CD8 T_FR_ between day 0 and day 2 in mock-spinoculated cultures (S2 Fig). Tim-3 is expressed on a significantly greater percentage of CD8 T_FR_ compared to CD8 conv in all culture conditions (i.e., mock-, X4-, and R5-spinoculated cultures) and there are no differences related to HIV infection (Fig 1C). Perforin expression is lower in CD8 T_FR_ compared to CD8 conv in all culture conditions, and is not altered by spinoculation (Fig 1D). The percentage of IL-10+ CD8 T_FR_ is significantly higher following both X4- and R5-spinoculation compared to mock-spinoculated cultures, and is significantly higher than the percentage of IL-10+ CD8 conv in each condition (Fig 1E). X4- and R5-spinoculation leads to significantly higher percentages of IL-15R+ CD8 T_FR_ compared to mock spinoculation, and there are greater percentages of IL-15R+ CD8 T_FR_ compared to IL-15R+ CD8 conv after HIV spinoculation (Fig 1F). The percentage of GITR+ CD8 T_FR_ does not increase after HIV spinoculation, but the frequency of GITR+ CD8 T_FR_ is greater than the frequency of GITR+ CD8 conv in all conditions (Fig 1G). CD122 is expressed on a greater percentage of CD8 T_FR_ after X4-spinoculation compared to mock-spinoculation, and is expressed on a greater percentage of CD8 T_FR_ compared to CD8 conv in all conditions (Fig 1H). There are not high levels of or significant differences in expression of the exhaustion molecule PD-1 or the inhibitory CD4 Treg receptor CTLA-4 between CD8 T_FR_ and conventional CD8 T cells (S3A Fig). Further, a majority of CD8 T_FR_ have an effector memory as opposed to central memory profile, and express more IL-10, CD122, and GITR and less perforin than conventional CD8 T cells regardless of effector or central memory cell designation (S3B Fig).

### The majority of CD8 T cells with a follicular homing phenotype are CD8 T_FR_

**Fig 2:**
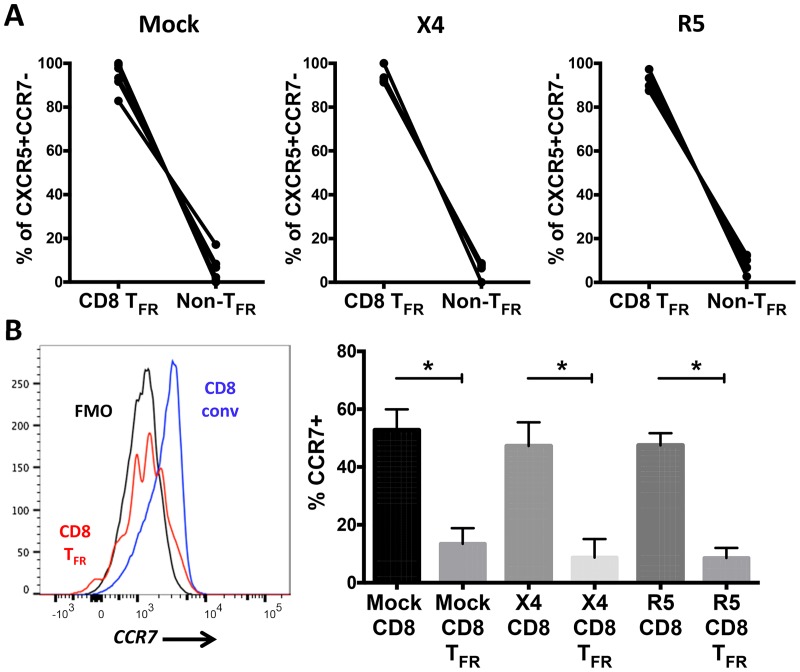
Most human tonsil follicular homing CD8 T cells are CD8 T_FR_. Disaggregated tonsil cell cultures were mock-spinoculated or spinoculated with X4- or R5-tropic HIV and cultured for 2 days (n = 6). (A) Of the viable CD3+CD8+ population expressing the follicular phenotype CXCR5+CCR7-, the percent CD44^hi^ (CD8 T_FR_) and all other CD3+CD8+ (CD8 conv) in mock- and HIV-spinoculated cultures is shown. (B) The percent CCR7 expression on CD8 T_FR_ (red) and CD8 conv (blue) compared to an FMO control (black). Graphs depict median and range. Statistical significance was determined by non-parametric one-way ANOVA (Friedman test) and is displayed as * = p<0.05.

Downregulation of CCR7, the extrafollicular retention molecule, combined with upregulation of CXCR5, the follicular homing molecule, is crucial for migration of CD4 T cells into follicles [34]. It has previously been shown in human tonsils that CXCR5+CD8+ T cells were located in the follicles of human tonsils by immunofluorescent tissue staining [35]. We determined the phenotype of CD8 T cells expressing the follicular homing phenotype CXCR5+CCR7- after mock-, X4-, and R5-spinoculation of human tonsil cells. A median of more than 90% of CXCR5+CCR7- cells are CD8 T_FR_ in all conditions whereas only a minority of CD8 conv exhibit a follicular homing phenotype (Fig 2A). We further analyzed CCR7 expression on both CD8 T_FR_ and CD8 conv. The percent of CCR7 positive cells is significantly higher in CD8 conv than CD8 T_FR_ (Fig 2B).

### CD8 T_FR_ inhibit T_FH_ and IgG production in HIV infection *ex vivo*

**Fig 3:**
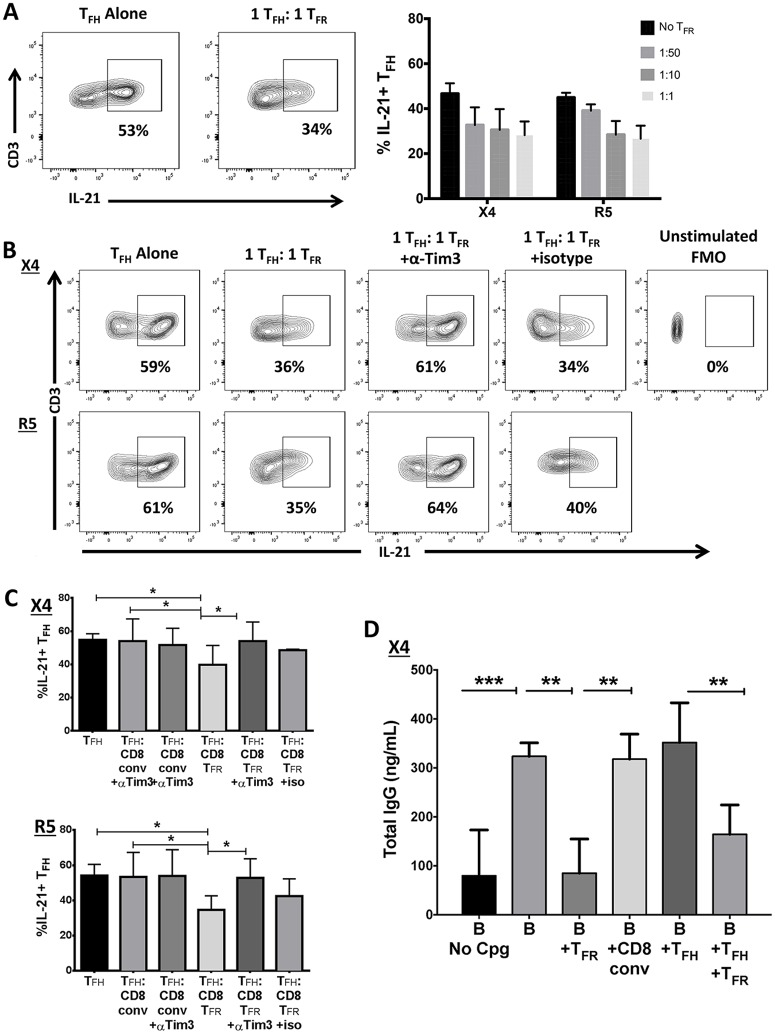
Human tonsil CD8 T_FR_ inhibit T_FH_ and GC B cell function. Tonsil cells were sorted to isolate CD8 T_FR_, CD8 conv, CD3+CD8-CXCR5+ T_FH_, and CD19+CD38+ GC B cells and X4- or R5-spinoculated. Cells were then co-cultured at indicated ratios for 2 days and analyzed. (A) IL-21 production by T_FH_ with increasing (left to right) number of CD8 T_FR_ (n = 4). (B) Representative examples from X4- and R5-spinoculations showing IL-21 production by T_FH_ alone, 1:1 with CD8 T_FR_, 1:1 with CD8 T_FR_ and anti-Tim3 antibody (500 ng/μl; right panels), and 1:1 with CD8 T_FR_ and an isotype control antibody (500 ng/μl). (C) Results from a total of 6 tonsils (isotype n = 3) as described in B. (D) IgG production in X4-spinoculated cultures with 2.5 μg/mL CpG-B stimulation in CD8 T_FR_, T_FH_, and B cell co-cultures as measured by ELISA. All co-cultures are 1:1 (n = 7). Statistical significance was determined by non-parametric Wilcoxon matched-pairs tests (B) or one-way ANOVA (Friedman test, C) and is displayed as * = p<0.05, ** = p<0.01 and *** = p<0.001.

We next investigated whether CD8 T_FR_ are able to suppress the GC reaction by inhibiting T_FH_ proliferation and cytokine production, B cell function, or both. T cell proliferation is dependent on IL-2, and IL-21 production by T_FH_ is critical for B cell affinity maturation in the GC [36]. We therefore determined whether CD8 T_FR_ inhibit IL-2 and IL-21 production by T_FH_. As whole tonsil cells were spinoculated for the data generated in Figs 1 and 2, all sorted populations were spinoculated to mirror previous conditions and allow exposure to HIV. We find that CD8 T_FR_ inhibit both IL-2 (S4A Fig) and IL-21 (Fig 3A) production by T_FH_ in sorted X4- and R5-spinoculated cultures. This inhibition is dose-dependent on the ratio of CD8 T_FR_ to T_FH_ and does not occur when T_FH_ are cultured with CD8 conv (S4B Fig). To investigate potential mechanisms regulating IL-21 production in T_FH_, we blocked Tim-3 in 1:1 CD8 T_FR_:T_FH_ co-cultures. Blockade of Tim-3 abrogates the effect of CD8 T_FR_ and preserves the percent of IL-21+ T_FH_ at levels similar to T_FH_ cultured alone (representative examples: Fig 3B, all data and 3C), but this is not observed in T_FH_ and CD8 T_FR_ co-culture with isotype control antibodies (Fig 3B and 3C) or T_FH_ and CD8 conv co-cultures (S4C Fig). There is not a consistent trend in alteration of T_FH_ proliferation rates, as determined by proliferation dye dilution, in the presence of CD8 T_FR_ when cultured at a 1:1 ratio (S5 Fig).

Next, we determined if CD8 T_FR_ influence total IgG production in tonsil cultures. We cultured isolated GC B cells (CD19+CD38+) with CD8 conv, CD8 T_FR_, and/or T_FH_ at equal ratios, mock- or X4-spinoculated the cells, and stimulated them with CpG-B for 6 days. There is not increased IgG production in T_FH_ and B cell co-cultures compared to B cells alone, likely due to the use of CpG-B that activates B cells directly through TLR9. In X4-spinoculated experiments, adding CD8 T_FR_ to B cells alone or to B cells and T_FH_ leads to decreased IgG production, however, adding CD8 conv to B cells has no effect on IgG production (Fig 3D).

### CD8 T_FR_ modestly reduce T_FH_ permissivity to HIV

**Fig 4:**
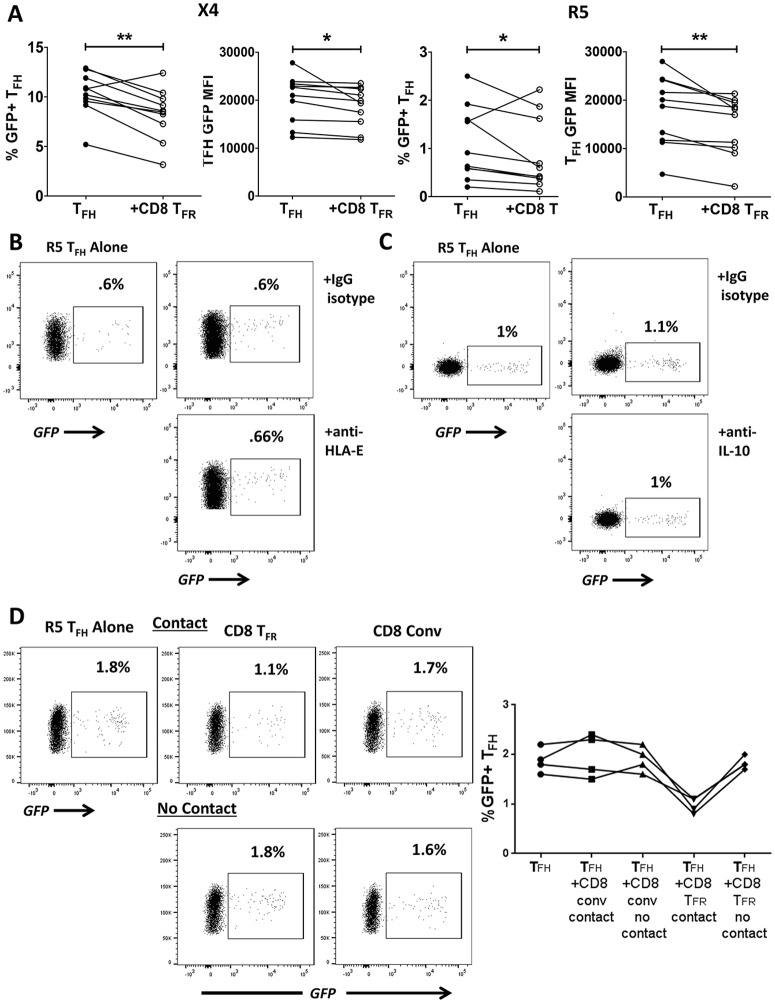
Human tonsil CD8 T_FR_ downregulate productive infection in T_FH_ in a contact-dependent manner. Tonsil cells were spinoculated with X4- or R5-tropic GFP-reporter HIV. T_FH_ were cultured alone or at a 1:1 ratio with CD8 T_FR_ for 2 days and analyzed by flow cytometry (n = 10). (A) Percent GFP+ T_FH_ and T_FH_ GFP MFI. (B) The percent R5-GFP+ T_FH_ when co-cultured with CD8 T_FR_ and either isotype control antibody or HLA-E blocking antibody (500 ng/μl, n = 2). (C) The percent R5-GFP+ T_FH_ when co-cultured with CD8 T_FR_ and either isotype control antibody or IL-10 blocking antibody (ng/μl, n = 2). (D) The percent R5-GFP+ T_FH_ when co-cultured in contact with or not in contact with CD8 T_FR_ and conventional CD8 (n = 4). Statistical significance was determined by Wilcoxon matched-pairs tests and is displayed as * = p<0.05 and ** = p<0.01.

As HIV replication is highly concentrated within T_FH_ *ex vivo* [6] and *in vivo* [1, 4, 5], we next investigated the effect of CD8 T_FR_ on HIV replication in *ex vivo* infected tonsillar T_FH_ using GFP-reporter viruses. Both the percentage of GFP+ T_FH_ and the MFI of GFP is modestly reduced when sorted T_FH_ are co-cultured 1:1 with CD8 T_FR_ (Fig 4A) following either X4- or R5-spinoculation. Co-culture with CD8 conv, on the other hand, does not alter HIV replication in T_FH_ (S6 Fig).

We next investigated potential mechanisms by which CD8 T_FR_ reduce HIV replication in T_FH_. In mice, CD8 T_FR_ inhibit T_FH_ responses via Qa-1 [19], the mouse equivalent of the HLA-E molecule. To determine whether this mechanism restricts viral replication in T_FH_, we added HLA-E blocking antibodies to co-cultures. Blocking HLA-E interactions in 1:1 CD8 T_FR_ and T_FH_ co-cultures has no effect on viral replication (Fig 4B). HIV replication has also been shown to be inhibited by IL-10 in PHA-blasted PBMCs and cell lines [37]. Nevertheless, we do not observe differences in viral replication in co-cultures of CD8 T_FR_ and T_FH_ with addition of IL-10 neutralizing antibodies (Fig 4C). However, inhibition of HIV replication in T_FH_ by CD8 T_FR_ is contact-dependent, as inhibition is not observed when cells are separated by transwell membranes in 4 separate experiments (Fig 4D).

### CD8 T_FR_ induce apoptosis in T_FH_ via HLA-E

**Fig 5:**
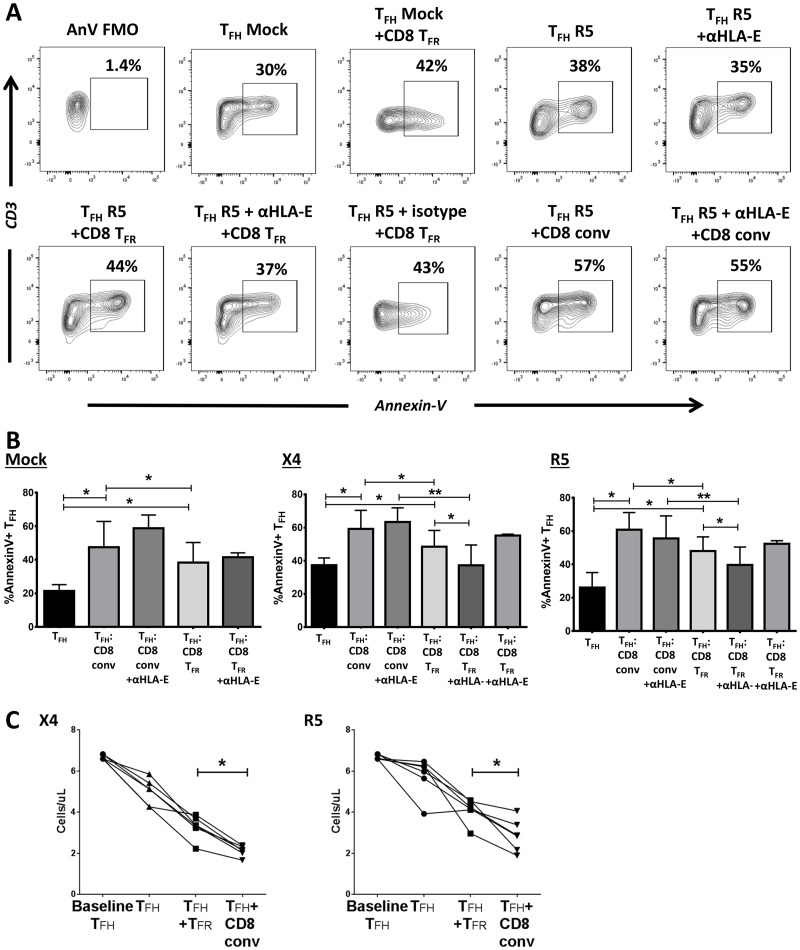
CD8 T_FR_ induces T_FH_ apoptosis via HLA-E. Human tonsil cells were sorted to isolate CD8 T_FR_, CD8 conv, and T_FH_, spinoculated with R5-tropic HIV, and cultured for 2 days. (A) Representative flow plots showing the percent of Annexin-V+ T_FH_ in co-culture at 1:1 ratio with CD8 T_FR_ or CD8 conv 2 days after R5-spinoculatoin. (B) Results from a total of 6 tonsil for mock-, X4-, and R5-spinoculation (isotype n = 3) in A. Co-cultures were also performed with HLA-E blocking antibody or isotype controls (500 ng/μl). (C) Number of T_FH_ per microliter on day 0 and day 2 when cultured alone (circle, triangle), 1:1 with CD8 T_FR_ (square), or 1:1 with conventional CD8 T cells (upside down triangle) (n = 6). Statistical significance was determined by Wilcoxon matched-pairs tests and is displayed as * = p<0.05 and ** = p<0.01.

As CD8 T_FR_ are located within the follicle, their ability (or lack thereof) to kill T_FH_ is important for understanding their role in the follicular concentration of HIV replication. In mice, CD8 T_FR_ are able to recognize and kill autoreactive T_FH_ via Qa-1 interaction [19]. Therefore, we wanted to determine if HLA-E, the human equivalent of Qa-1, similarly allowed CD8 T_FR_ to kill T_FH_ in tonsil cultures. As tonsil cells are from HIV-uninfected subjects, we did not expect to see virus-specific killing and looked broadly at T_FH_ apoptosis rates using Annexin-V. We investigated the rate of apoptosis in mock-, X4-, and R5-spinoculated T_FH_ alone or in co-culture with CD8 T_FR_ or CD8 conv. After cell populations were sorted, all cells were spinoculated, cultured together at 1:1 ratios for 2 days, and stained for Annexin-V to determine apoptosis rates of T_FH_. When cultured at a ratio of 1:1 with T_FH_, CD8 T_FR_ induce less apoptosis in T_FH_ than CD8 conv (representative examples: Fig 5A, cumulative data and 5B). We find that the rate of apoptosis in T_FH_ is reduced further by blocking HLA-E in 1:1 CD8 T_FR_ and T_FH_ co-cultures (Fig 5A and 5B). Blocking HLA-E in CD8 conv/T_FH_ co-cultures has no effect (Fig 5A and 5B). The use of count beads during co-culture analyses shows that conventional CD8 T cells reduce the number of T_FH_ in both X4- and R5-spinoculation, while co-culturing T_FH_ with CD8 T_FR_ reduces T_FH_ numbers to a lesser extent (Fig 5C).

### CD8 T_FR_ increase and have a distinct phenotype during chronic SIV infection

**Fig 6:**
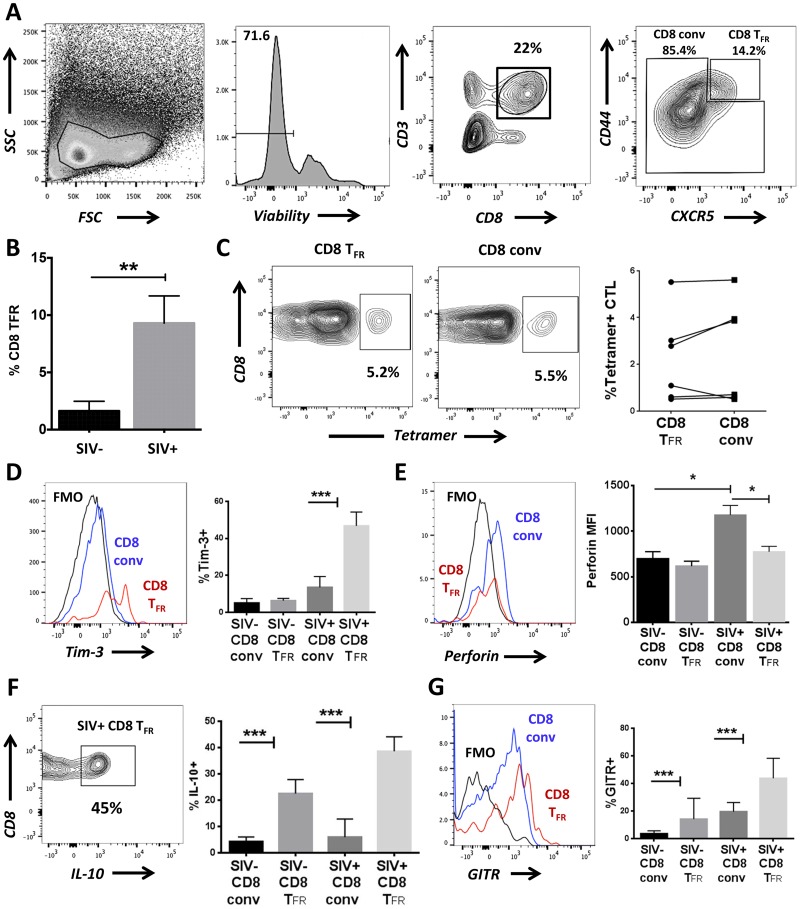
CD8 T_FR_ are higher in SIV-infected than uninfected rhesus macaques. Disaggregated cells from lymph nodes and spleen of SIV-uninfected (n = 6) and SIV-infected (n = 6) rhesus macaques were analyzed for CD8 T_FR_ by flow cytometry. (A) Flow gating strategy to determine viable CD8 T_FR_ (CD3+CD8+CXCR5^hi^CD44^hi^) and CD8 conv. (B) Percent CD8 T_FR_ in SIV-uninfected compared to SIV-infected rhesus macaques. (C) Percent of SIV-Gag tetramer+ CD8 T_FR_ compared to CD8 conv. CD8 T_FR_ and CD8 conv from SIV-uninfected and SIV-infected rhesus macaques were analyzed for percent or MFI expression of (D) Tim-3, (E) perforin, (F) IL-10, and (G) GITR. Graphs depict median and range. Statistical significance was determined by non-parametric one-way ANOVA (Friedman test) and is displayed as * = p<0.05 and *** = p<0.001.

Next, we investigated the effects of SIV infection on the frequency of CD8 T_FR_ in secondary lymphoid tissues *in vivo*. Using the same phenotype as in 1A for human tonsils, we define CD8 T_FR_ as viable CD3+CD8+CXCR5^hi^CD44^hi^ cells (Fig 6A). We compared the percentage of CD8 T_FR_ in disaggregated spleen and lymph nodes from chronically SIV-infected rhesus macaques to those from SIV-uninfected controls and find that percentages of CD8 T_FR_ are increased a median of 4-fold during SIV infection compared to uninfected controls (Fig 6B). Additionally, we determined whether the proportion of SIV-specific cells differed among CD8 conv and CD8 T_FR_. We find that the frequency of SIV-Gag specific CD8 T_FR_ is similar to that of CD8 conv (Fig 6C). There were positive, but statistically insignificant correlations between the percent CD8 T_FR_ or percent tetramer+ CD8 T_FR_ and viral loads (S7 Fig).

We determined the expression of key CD8 regulatory molecules on CD8 T_FR_ in rhesus macaques as done with human tonsils in Fig 2. In chronically SIV-infected animals, there is a significantly greater frequency of Tim-3+ CD8 T_FR_ compared to CD8 conv (Fig 6D). Perforin expression on CD8 conv is significantly greater in SIV-infected compared to SIV-uninfected animals, while CD8 T_FR_ do not display this trend and have significantly less perforin than CD8 T conv in SIV-infected animals (Fig 6E). The percentage of CD8 T_FR_ that produce IL-10 is higher than CD8 conv in both SIV-uninfected and chronically SIV-infected macaques. Furthermore, the percentage of CD8 T_FR_ that produce IL-10 is higher in SIV-infected compared to uninfected animals (Fig 6F). The percentage of IL-10 producing CD8 conv is similar in uninfected and SIV-infected macaques (Fig 6F). The percentage of Galectin-9 (Gal-9)+ CD8 T_FR_ tends to be higher than the percentage of Gal-9+ CD8 T conv in SIV-infected animals, but differences were not statistically significant (S8 Fig), while GITR is significantly increased on CD8 T_FR_ in both SIV-uninfected and SIV-infected animals (Fig 6G).

### The majority of follicular CD8 T cells in chronic SIV infection are CD8 T_FR_

**Fig 7:**
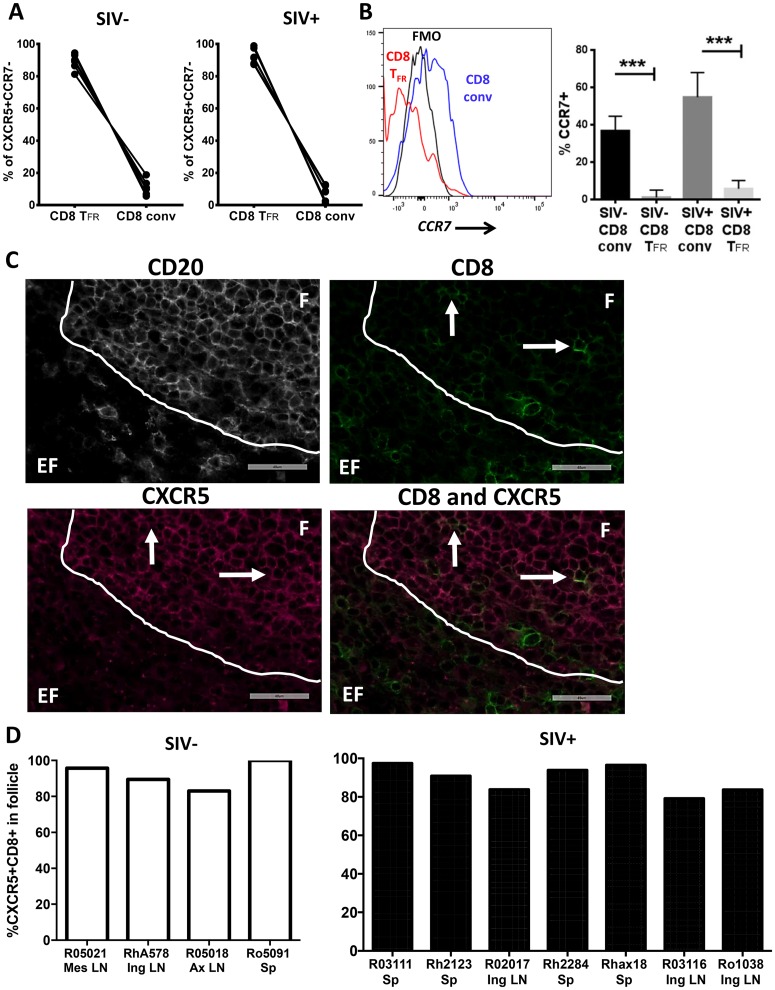
Most rhesus macaque CXCR5+CCR7- CD8 T cells are CD8 T_FR_. Disaggregated cells from lymph node and spleen of SIV-uninfected (n = 6) and SIV-infected (n = 6) rhesus macaques were analyzed by flow cytometry. (A) Percentages of CD8 T_FR_ and CD8 conv from all CD3+CD8+CXCR5+CCR7- are shown for SIV-uninfected and SIV-infected animals. (B) The percent CCR7+ cells in CD8 T_FR_ and CD8 conv cell populations(C) Representative images of rhesus macaque spleen tissue staining. CD20 staining was performed to determine follicular (F) and extrafollicular (EF) regions. (D) The percentage of CXCR5+ CD8 T cells in follicular and extrafollicular regions as determined from images similar to C. Left plot (white bars) show uninfected rhesus macaques, right plot (black bars) show chronically infected rhesus macaques. LN = lymph node, Mes = mesenteric, Ing = inguinal, Ax = axial, and Sp = spleen. Graphs depict median and range. Statistical significance was determined by non-parametric one-way ANOVA (Friedman test) and is displayed as *** = p<0.001.

As shown previously for human tonsils in Fig 2, we determined the phenotype of CD8 T cells expressing the follicular homing profile CXCR5+CCR7- in lymph node and spleen of uninfected or chronically SIV-infected rhesus macaques. The vast majority of CXCR5+CCR7- cells are CD8 T_FR_ (median 89% of uninfected, 93% of SIV-infected) whereas only a minority of CD8 conv exhibit a follicular homing phenotype (Fig 7A). We further analyzed CCR7 expression on both CD8 T_FR_ and CD8 conv. The percent of CCR7 positive cells is significantly higher in CD8 conv than CD8 T_FR_ in both SIV-infected and uninfected rhesus macaques (Fig 7B). Further, we performed immunofluorescent staining of lymph node and spleen tissue sections to determine the precise location of CXCR5+ CD8 T cells. As shown in representative images (Fig 7C), the follicle was defined as CD20+ (white) and the number of CD8 T cells (green) expressing CXCR5 (red) were counted as either inside the follicle or in the extrafollicular zone. Most CXCR5+ CD8 T cells, a median of 91% in chronically infected rhesus macaques and a median of 93% in uninfected rhesus macaques, were found inside of the follicle (Fig 7D). In light of findings above indicating that the vast majority of CXCR5+CD8+ T cells exhibit a CD8 T_FR_ phenotype, it is reasonable to conclude that most cells with that phenotype reside in the follicle.

### CD8 T_FR_ inhibit T_FH_ function via Tim-3 and induce T_FH_ apoptosis via HLA-E in chronic SIV infection

**Fig 8:**
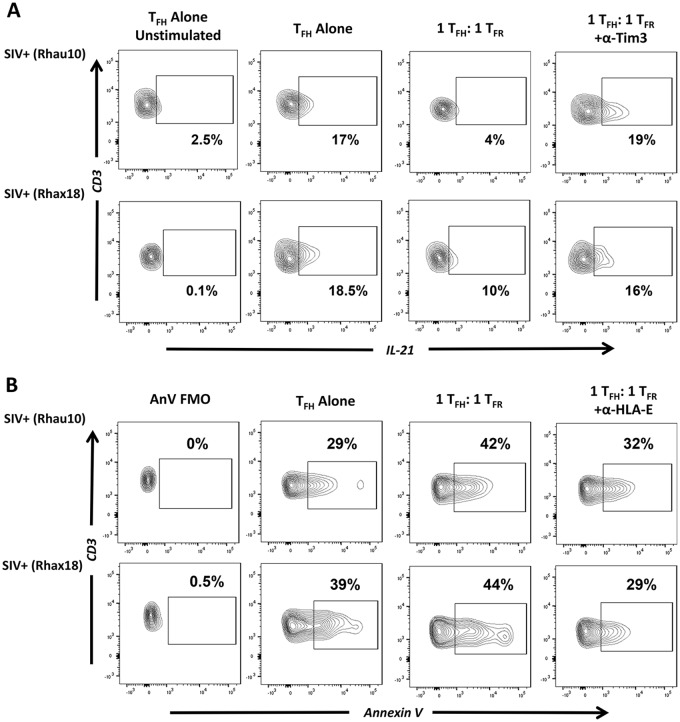
CD8 T_FR_ suppress IL-21 production in T_FH_ via Tim-3 and induce apoptosis via HLA-E in SIV-infected rhesus macaques. Disaggregated cells from lymphoid tissues of SIV-infected rhesus macaques (n = 2) were sorted for T_FH_ and CD8 T_FR_ and co-cultured at a 1:1 ratio for 2 days with or without HLA-E blocking antibody and analyzed by flow cytometry. (A) Flow gating showing the percent T_FH_ producing IL-21, and (B) percent T_FH_ expressing Annexin-V (n = 2).

Utilizing isolated CD8 T_FR_ and T_FH_ from chronically SIV-infected rhesus macaques, we determined if CD8 T_FR_ regulated T_FH_ function or apoptosis rates. Using disaggregated lymph node cells from 2 SIV-infected animals, we find that IL-21 production by T_FH_ is inhibited when cultured with equal numbers of CD8 T_FR_ (Fig 8A) but not CD8 conv (S9A Fig). IL-21 production by T_FH_ is not reduced in co-cultures treated with Tim-3 neutralizing antibodies in culture (Fig 8A). The percentage of apoptotic T_FH_, determined by Annexin-V staining, is increased when T_FH_ are cultured with equal numbers of CD8 T_FR_ (Fig 8B). When HLA-E interactions are blocked with neutralizing antibodies in T_FH_: CD8 T_FR_ co-cultures, the percentage of Annexin-V+ T_FH_ decreases (Fig 8B) but not when cultured with CD8 conv (S9B Fig).

## Discussion

This is the first study to examine the role of CD8 T_FR_ in the context of HIV and SIV infection. We determined that the majority of follicular CD8 T cells in human tonsils, as well as in secondary lymphoid tissues from rhesus macaques, exhibit a CD8 T_FR_ phenotype. Of all the CD8 T cells expressing the follicular homing profile CXCR5+CCR7-, 90% or more of these cells are CD8 T_FR_ in both human tonsils and rhesus macaque lymphoid tissues. Furthermore, their percentage is higher in lymph nodes and spleens of chronically SIV-infected rhesus macaques compared to uninfected macaques. A greater percentage of CD8 T_FR_ express IL-10 and CD8 T_FR_ express relatively lower levels of perforin compared to CD8 conv. In *ex vivo* HIV spinoculation and chronic SIV infection, CD8 T_FR_ inhibit IL-21 production by T_FH_ and IL-21 production is rescued by Tim-3 blockade. Further, CD8 T_FR_ inhibit IgG production by B cells in *ex vivo* HIV infection. Additionally, CD8 T_FR_ significantly, albeit modestly, reduce T_FH_ permissivity to HIV *ex vivo* through contact-dependent mechanisms. Compared to CD8 conv, CD8 T_FR_ induce a low level of T_FH_ apoptosis. Blocking HLA-E interaction in CD8 T_FR_ and T_FH_ co-cultures further reduces T_FH_ apoptosis rates. Collectively, these studies demonstrate that CD8 T_FR_ constitute the majority of CD8 cells in B cell follicles and contribute to impaired GC function in lentiviral infections.

Multiple factors may account for the inability of CD8 T cells to control HIV and SIV replication in the follicles. Heightened permissivity of T_FH_ [6] combined with a paucity of virus-specific CTL in B cell follicles may foster virus replication at those sites [2, 9, 38]. Previous work in rhesus macaques has shown that CD8 is downregulated on SIV-specific cells entering the follicle [39] and CD8 downregulation leads to impairments of CTL cytokine production and proliferation [40]. It has been proposed that virus-specific CD8 T cells are exhausted in chronic infection [41, 42] or that virus escape mutations prevent virus-specific CD8 T cells from recognizing infected cells [43, 44], but these hypotheses do not fully account for the follicular concentration of HIV replication. Here, we show that most of the follicular CD8 T cells in humans and rhesus macaques are CD8 T_FR_, CD8 T_FR_ induce T_FH_ apoptosis at lower rates than CD8 conv, and blocking HLA-E recognition further reduces CD8 T_FR_–mediated apoptosis in T_FH_. We also demonstrate that CD8 T_FR_ produce a modest, but statistically significant reduction in HIV replication in human tonsil cells infected *ex vivo*. In the LCMV-infected mouse model, Leong et. al. [45] recently found that mice reconstituted with CXCR5+CD8 LCMV-specific T cells had 50% fewer LCMV-producing T_FH_ compared to mice reconstituted with CXCR5-CD8 T cells. Additionally, this study showed that CXCR5+CD8 LCMV-specific T cells express less perforin and are less efficient killers of LCMV-infected cells than their CXCR5- counterparts *ex vivo* [45]. These findings are consistent with our findings in the *ex vivo* HIV infection model and in chronic SIV infection that CD8 T_FR_ express less perforin than conventional CD8 T cells in both human tonsils and rhesus macaque lymphoid tissues, and that they induce modest reductions in HIV replication and T_FH_ apoptosis *ex vivo*. In marked contrast, He et. al. [46], reported profound reductions between 100- and 1,000-fold in splenic viral loads in the presence of CXCR5+CD44^hi^CD8 T cells compared to CXCR5-CD44^hi^CD8 T cells using the LCMV-infected mouse model. Reasons for the discrepancy between this study and that of Leong et. al. are not clear, as similar markers were used to define follicular populations of CD8 T cells in their studies, and these were the same markers that we used to define T_FR_ CD8 populations in our study. An important area of future investigation will be to determine if CD8 T_FR_ killing capacity can be boosted numerically and functionally to reduce follicular HIV replication *in vivo*.

Mechanisms that underlie the modest reduction in HIV replication induced by CD8 T_FR_ in our studies are not clear. This could be due to general immune suppressive mechanisms, such as lowering T_FH_ activation levels, or alternatively through cell killing. Previous work has shown that CD4 Treg suppressed HIV replication in other CD4 T cell populations through contact-dependent inhibitory mechanisms rather than inducing cytotoxicity [47]. CD4 and CD8 regulatory cell subsets likely have complex roles in HIV infection, as evidenced by their ability to inhibit effector functions, and also reduce HIV replication. Further, CD8 T_FR_ may promote the follicular HIV reservoir by inhibiting T_FH_ IL-21 production, as it has been shown that ART combined with IL-21 therapy results in lower viral loads and lower intestinal cell-associated SIV DNA in rhesus macaques when compared to ART alone [48]. Determining at which point in the virus life cycle immune regulatory mechanisms inhibit viral replication could provide useful information to promote a balance of effector cell function and inhibition of HIV replication. As CD8 T_FR_ display an ability to interfere with HIV replication *ex vivo*, future studies will be needed to determine if CD8 T_FR_ inhibit viral replication or eliminate virus producing cells, as these both have distinct implications for HIV cure strategies.

In this study, we show that CD8 T_FR_ expand in chronic SIV infection. However, we do not see this expansion in our *ex vivo* tonsil model after 2 days of infection. A likely explanation for this discrepancy is that the expansion of CD8 T_FR_ is a result of antigen-specific expansion in chronic HIV or SIV infection, and therefore not recapitulated in an acute infection model. This is supported by our data that show CD8 T_FR_ are SIV-specific at a similar frequency as CD8 conv. We have previously reported that total levels of CD8 expression in lymph node follicles increase in chronically HIV-infected subjects compared to seronegative individuals [1]. The present study demonstrates that the increase of follicular CD8 T cells in chronic HIV infection is likely due to an expansion of CD8 T_FR_. Therapies aimed at generating protective virus-specific CTL in patients will need to verify that cytotoxic responses, as opposed to regulatory responses, are being produced in the follicle.

An interesting question remaining is how CD8 T_FR_ are generated during HIV and SIV infection. It has been suggested that persistence of antigen is the cause of functional impairment of HIV-specific effector responses [49]. T_FH_ are expanded in HIV [4, 50] and SIV [3] infection, upregulate HLA-E upon HIV infection [51], and constant antigenic stimulation occurs in the follicle and GC [52, 53]. It is possible that CD8 T_FR_ are generated through contact with HLA-E on T_FH_ as they enter follicular and GC regions. These interactions could convert effective CTL into CD8 T_FR_ and allow for HIV persistence in the follicle. Further investigation as to whether CD8 T_FR_ migrate into the follicle or are converted from precursors after migrating into the follicle will be an important aspect of potentially manipulating or preventing this response to promote cytotoxicity.

It was shown that CD8 T cells from IL-15 knock-out mice lose suppressive capabilities [19]. Importantly, CD8 Treg from these mice were unable to suppress T_FH_ number, GC expansion, and IgG production. Interestingly, expansion and cytotoxicity of CD8 T cells is impaired in IL-21 knockout mice, but the combination of IL-21 and IL-15 synergistically improves CD8 effector function [54]. Additionally, IL-15 has been shown to have therapeutic potential as an adjuvant in HIV vaccines [55, 56] and in restoring CTL function [57, 58]. Here, we find that CD8 T_FR_ have elevated IL-15R expression and suppress T_FH_ IL-21 production in HIV infection *ex vivo*. As CD8 T_FR_ have access to HIV-infected T_FH_ in the GC, it will be interesting to determine if the use of IL-15 and IL-21 in HIV therapy increases CD8 T_FR_ cytotoxic function and promotes the elimination of HIV-infected T_FH_.

In chronic HIV infection expression of exhaustion markers such as PD-1 [42] and Tim-3 [29] correlate with diminished cytotoxic capacity. The inhibitory cytokine IL-10 was specifically produced by Tim-3+PD-1+ CD8 T cells after stimulation with LCMV peptides, suggesting Tim-3 is a potential target to limit regulatory cytokine production and promote CTL function. We find that inhibition of Tim-3 prevents impairment of T_FH_ function by CD8 T_FR_. Thus, Tim-3 blockade could provide a new mechanism to reverse CD8 regulatory functions and perhaps boost T_FH_ function. Further studies addressing the inhibitory mechanisms of CD8 T_FR_ and their role in T_FH_ impairment would be useful to determine how to boost the high quality response of T_FH_ to HIV vaccines.

Previous work in mice showed that CD8 T_FR_ do not directly suppress antigen-specific antibody production [19], however CD8 T cells have been shown to directly suppress non-specific antibody responses in other models [59]. A previous study found that both CXCR5-CD8+ and CXCR5+CD8+ human tonsil cells supported IgG production by CD19+ B cells in uninfected, unstimulated conditions [35]. Here we find that CD8 T_FR_ are able to directly suppress total IgG production by B cells in a stimulated, HIV-spinoculated culture. This is not observed when B cells are cultured with CD8 conv, indicating a unique effect of CD8 T_FR_ on GC B cells. A previous study using human tonsil cells showed that CD4 Treg were also able to suppress IgG production by B cells [60]. Thus, the mechanism for direct suppression of B cells could be a general feature of all regulatory T cell subsets, through common expression of Tim-3 or GITR, or a mechanism unique to CD8 T_FR_ such as sequestration of IL-15 since IL-15 knock-out mice have been shown to produce less IgG [61]. Further, it is unknown at this time if CD8 T_FR_ can suppress antibody production elicited by T_FH_, but it is likely as CD8 T_FR_ strongly inhibited IL-21 production by T_FH_. Future studies to determine whether CD8 T_FR_ suppress HIV-specific antibody responses are warranted. CD8 T_FR_ suppression of B cell function could potentially account for why HIV-infected individuals have poor responses to T cell-independent vaccines, such as the polysaccharide vaccines against *pneumococcus* [62] and *S*. *typhi* [63]. A better understanding of the role of CD8 T_FR_ in generation of antigen-specific antibody responses could lead to innovative strategies to improve vaccine responses in HIV-infected individuals.

Reduction of pro-inflammatory responses prior to high-dose SIV challenge prevented mucosal transmission [64], suggesting regulatory immune cell function could be an important aspect of reducing HIV infection and replication. Interestingly, the induction of immune tolerance via CD8 Tregs was shown to have a protective role in rhesus macaques prior to SIV challenge [65]. Specifically, non-classical MHC-Ib/E restricted CD8 T cells from SIV-immunized animals inhibited CD4 T cell activation and SIV replication within autologous CD4 T cells infected *ex vivo*, but only if the CD8 T cells were added within 48 hours [65]. We similarly observe that CD8 T_FR_ reduce HIV replication in T_FH_ *ex vivo* after 2 days. Taken together with our results that CD8 T_FR_ induce less apoptosis in T_FH_ than conventional CD8 T cells, we hypothesize that CD8 T_FR_ would limit cellular activation and HIV replication within T_FH_ on a per cell basis whereas CTL would reduce the number of HIV-infected T_FH_. However, these data were obtained in an *ex vivo* infection model and lack HIV-specific responses, so further studies *in vivo* would be necessary to determine if CD8 T_FR_ and CTL differentially affect the follicular HIV reservoir. Although we observe only modest reductions in HIV replication in tonsil cells infected *ex vivo*, our data supports the notion that inflammation and cellular activation promote HIV infection and replication. However, regulation of infected cells may have drawbacks, as opposed to benefits prior to infection. An in-depth analysis is necessary to determine if immune regulatory activity on infected cells is inhibiting replication pre- or post-integration and if this promotes formation of the latent HIV reservoir.

In this study, we find that most follicular CD8 T cells are CD8 T_FR_ and they potently impair T_FH_ and GC B cell responses. The heightened regulatory function and relative lack of cytolytic potential of CD8 T_FR_ could contribute to viral persistence in the follicle and impairments of humoral immunity that are characteristic of HIV and SIV infection. This is a novel mechanism of regulation of humoral immunity and remains to be explored in the context of other human diseases. Development of therapies that block CD8 T_FR_ interaction with T_FH_ and GC B cells could lead to novel approaches to improve the quality of antibody responses in HIV infection.

## Materials and Methods

### Ethics statement

Human tonsils were obtained from the Colorado Children’s Hospital (Aurora, Colorado, USA) following routine tonsillectomy from individuals at low risk for HIV infection. Use of tonsil specimens for these studies was reviewed by the Colorado Multiple Institutional Review Board and determined to not constitute human subjects research (COMIRB approval no. APP001-1), in accordance with guidelines issued by the Office of Human Research Protections (http://www.hhs.gov/ohrp/policy/checklists/decisioncharts.html), and consequently, informed consent was not required. All research involving human subjects conformed to the principles set forth in the Declaration of Helsinki and was approved by the Colorado Multiple Institutional Review Board. Rhesus macaques were cared for according to the guidelines of the Animal Welfare Act and the NIH for housing and care of laboratory animals. Animal experiments were approved by the Institutional Animal Care and Use Committee of the University of Wisconsin (IACUC; protocol G00632). Procedures were performed to ensure that discomfort was limited to that unavoidable in the conduct of the research plan. Animals were housed at the Wisconsin National Primate Research Center (WNPRC), which is accredited by American Association of Accreditation of Laboratory Animal Care (Animal Welfare Assurance No. A3368-01). Sedatives were applied as necessary for blood and tissue collections and analgesics were used when determined appropriate by veterinary medical staff. Animals were fed standard monkey chow twice daily. Pain, distress, animal behavior, food and drink consumption was monitored and adjustments were made as necessary. SIV-infected rhesus macaques were singly housed, but had visual and auditory contact with at least one social partner, permitting the expression of non-contact social behavior. Animals had access to more than one category of enrichment at WNPRC. The IACUC proposal included a written scientific justification for any exclusion from some parts of the enrichment plan. Research-related exemptions are reviewed at least annually.

### Macaques

Lymph nodes and spleen were obtained from 6 SIVmac239-infected and 6 uninfected Indian rhesus macaques (*Macaca mulatta*). Animals were infected either intravenously or intrarectally with SIVmac239, and had been infected from 12 to 241 weeks (median, 19.5 weeks) at the time that specimens were obtained. Plasma SIV RNA concentrations ranged from 3.78 to 6.45 log_10_ copies/ml (median, 5.45 log_10_ copies/ml), and CD4+ T cell counts ranged from 291 to 422 cells/mm^3^ (median, 362 cells/mm^3^). Of SIV-infected animals, 2 were female and ranged in age from 7 to 8 years old. Of SIV uninfected animals, 2 were female and ranged in age from 10 to 23 years old. Tissues were either shipped overnight on ice in cold RPMI 1640 and disaggregated, or disaggregated and cryopreserved at the Wisconsin National Primate Research Center and later shipped on liquid nitrogen to the University of Colorado.

### HIV reporter viruses and tonsil cultures

The HIV-1 NL4-3-based CXCR4 (X4)-tropic green fluorescent protein (GFP) reporter virus NLENG1-IRES [66] and the CCR5 (R5)-tropic GFP reporter virus NLYUV3-GFP [67] were used for tonsil cell infections. Virus stocks were prepared by transfecting 293T cells (ATCC) with either X4 or R5 plasmid constructs (Effectene, Qiagen 301425) in complete DMEM (DMEM + 10% FBS, pen/strep, and non-essential amino acids), collecting supernatants, and spinning at 800 *x g* to remove debris. Viral stocks were stored at -80°C prior to use. After disaggregation, 5 x 10^6^ tonsil cells were spinoculated with either GFP reporter virus or a mock spinoculation with an equal volume of complete DMEM for 2 hours at 1200 *x g* at room temperature. Cells were washed to remove unbound virus and media, and cultured for 2 days at 37°C with 5% CO_2_ in RPMI with 10% FBS, L-glutamine, and pen/strep (R10) at a density of 1.5 x 10^6^ cells/mL. Cells were collected and immediately processed for analysis by flow cytometry.

### Flow cytometry and antibodies

Cells were blocked for 20 minutes with 2% BSA in PBS at 4°C and then stained for 30 minutes at 4°C in the dark. The following anti-human conjugated antibodies were used: CD3-APCCy7-UCHT1 (Tonbo 25–0038), CD8-eVolve605-RPA-T8 (eBioscience 83–0088), CD19-FITC-SJ25C1 (Tonbo 35–0198), CD38-violetFluor450-HIT2 (Tonbo 75–0389), CXCR5-PE-MU5UBEE (eBioscience 18–9185), PD-1-APC EH12.2H7 (BioLegend 329908), GITR-PECy7-eBioAITR (eBioscience 12–5875), CD44-APC-IM7 (Tonbo 20–0441), Tim3-APC-344823 (R&D FAB2365A), IL-15R-FITC-eBioJM7A4 (eBioscience 11–7159), CD122-V421-Mikβ3 (BD 562887), PD-1-APC-EH12.2H7 (BioLegend 329908), CTLA-4-PECy5-BNI3 (BD 561717), CD62L-PE-SK11 (BD 341012), and CCR7-PECy7-3D12 (BD 557648). The same antibody panel was used for rhesus macaque cell staining with the exception of CD3 SP34-2 (BD 557757). All analyses were performed on Ghost Dye 510 (Tonbo 13–0870) negative cells. Cells were fixed with 2% paraformaldehyde. Fresh human tonsil cells were typically 70–90% viable after culture and cryopreserved rhesus macaque cells ranged from 40–80% viability after freeze/thaw. All antibodies were used at one test per 10^6^ cells. Data were acquired on a custom LSR II flow cytometer (Serial # H47100196, BD Immunocytometry System, San Jose, CA) with BDFACS Diva (v6.1) and with a configuration of 6 filters (755LP, 685LP, 670LP, 635LP, 600LP, 550LP, and 505LP) on a blue laser (488 nm), 6 filters (750LP, 690LP, 635LP, 595LP, 505LP, and 450/50) on a violet laser (405 nm), and 3 filters (755LP, 685LP, and 670/30) on a red laser (633 nm). FCS files were analyzed using FlowJo (v10.7, Tree Star, Ashland, OR).

### Rhesus macaque tetramer staining

Briefly, biotinylated MHC class I monomers were loaded with peptides (NIH Tetramer Core Facility) and converted to MHC tetramers with APC streptavidin (Prozyme PJ27S). The MHC class I monomer Mamu-A*001:01 molecule loaded with SIV Gag CM9 (CTPYDINQM) peptides was used. Cryopreserved disaggregated lymphoid tissue cells were thawed, and 1–2 x 10^6^ disaggregated cells were resuspended in 100 μL of tetramer staining buffer (5% fetal bovine serum in PBS with 0.06% sodium azide) and incubated with APC-labelled Gag CM9 tetramer concurrently with CXCR5-PE-MU5UBEE, CD8-eFlour605-RPA-T8, CD44-eFluor450-IM7, CD3-APC Cy7-SP34-2 and Ghost Violet 450 viability in the dark for 40 minutes at room temperature.

### Lymphoid tissue staining

SIV-uninfected (R05021 mesenteric lymph node, RhA578 inguinal lymph node, R05018 axial lymph node, and R05091 spleen) and chronically SIV-infected (R03111 spleen, Rh2123 spleen, R02017 inguinal lymph node, Rh2284 spleen, Rhax18 spleen, R02116 inguinal lymph node, and Ro1038 inguinal lymph node) rhesus macaque lymphoid tissues were analyzed as follows. Four micron frozen tissue samples were fixed in 1% paraformaldehyde and stained with Rabbit anti CD20 (Abcam, Cambridge, MA), Rat anti CD8 (Bio Rad, Hercules, CA) and mouse anti CXCR5 (NIH NHP Reagent Resource) followed by detection with AF647 Donkey anti rabbit, AF594 Goat anti Mouse (highly cross absorbed) and AF488 Goat anti rat (highly cross absorbed) (Thermo Fisher Scientific, Waltham, MA). Sections were counterstained with DAPI and imaged on a Leica IMI6000 inverted fluorescent microscope. Ten 40X images were analyzed using Qwin (Leica Microsystems) by first defining the follicular region and counting CD8+CXCR5+ cells within each region and calculating the frequency of cells both in and out of the follicle. An adjacent section was stained for CD20 only and visualized using Vector HP Immpress and Vector Red (Vector Laboratories, Burlingame, CA) and the total follicular and extrafollicular area determined using Qwin. The percentage of CD8+CXCR5+ cells in the follicle was determined based on the frequency of cells within the follicular and extrafollicular regions and the total areas of each region.

### Sorting and co-culture experiments

Human tonsil and rhesus macaque lymph node cells were sorted using a MoFlo Astrios EQ. Cells were sorted into T_FH_ (CD3+CD8-CXCR5+), conventional CD8 (CD3+CD8+CXCR5-), CD8 T_FR_ (CD3+CD8+CXCR5^hi^CD44^hi^), and GC B cell (CD19+CD38+) populations. After sorting, all tonsil cell subsets were spinoculated with X4- or R5-HIV GFP reporter viruses and cultured in R10. All cell populations were spinoculated to mirror whole tonsil cell cultures and allow for all cell populations to be exposed to virus. For measurements of T_FH_ cytokine production, T_FH_ were seeded at 1 x 10^5^ cells in a 24 well plate and CD8 T_FR_ were added at ratios of 1:1, 1:10, and 1:50. After 2 days, T_FH_ were stimulated to measure IL-2 and IL-21 by ICS. For blocking experiments, T_FH_ and CD8 T_FR_ or conventional CD8 were cultured at a 1:1 ratio in the presence of 500 ng/μL anti-Tim-3 (Biolegend 345003) antibodies or 500 ng/μL anti-HLA-E (Biolegend 342602) antibodies for 2 days. In transwell assays, 4 x 10^4^ T_FH_ were cultured in the bottom compartment and 4 x 10^4^ CD8 T_FR_ or conventional CD8 T cells were added to the upper compartment of 96-well permeable support plates (Corning CLS3386) and cultured for 2 days.

### ICS assays

After 2 days of culture, tonsil cells were stimulated with 50 ng/mL of phorbol 12-myristate 13-acetate (PMA, Sigma P8139) and 1 μg/mL of ionomycin (Sigma I3909) in the presence of protein transport inhibitor containing monensin (BD GolgiStop) for 5 hours. Cells were then harvested, blocked and stained for surface markers as above, and then fixed and permeabilized using BD CytoFix/Cytoperm kit (554714) according to manufacturers’ instructions. Cells were then stained at 4°C for 30 minutes and analyzed for IL-2-PE-MQ1-17H12 (BD 559334), IL-10-eFluor450-JES3-9D7 (eBioscience), IL-21-AF647-3A3-N2.1 (BD 560493), and perforin-FITC-Pf344 (Mabtech 3465–7). All cytokine analyses were normalized to a mock-spinoculated control that received monensin but was not stimulated.

### IgG ELISA

After sorting, 5 x 10^4^ GC B cells and CD8 T_FR_ T_FH_ were cultured in the presence or absence of T_FH_ at ratios of 1:1:1. Cells were co-cultured in R10 for 6 days in 96 well plates and treated with either 2.5 μg/mL of CpG-B or were unstimulated. Media was collected and spun at 5,000 *x* g to remove cellular debris and stored at -80°C. ELISAs were performed using the total IgG kit (Ready set go, eBioscience 88–50550) according to manufacturer’s instructions. Briefly, 96 well plates were coated overnight with anti-human IgG and culture supernatants were diluted at a 1:10 ratio for detection. Supernatants were incubated on pre-coated plates for 2 hours, washed, and incubated with HRP anti-IgG for 1 hour. After addition of HRP substrate, plates were analyzed on a plate reader at 450 nm and IgG calculated based on standards.

### Cell proliferation

T_FH_ (CD3+CD8-CXCR5+CD25-) were sorted and stained with proliferation dye (Cell Proliferation Dye eFluor670, eBioscience 65–0840) at a concentration of 0.5 μM. In a 96-well plate, pre-coated with 5 μg/mL anti-CD3 (Tonbo 40–0037) in PBS at 37°C for 2 hours, 10^4^ T_FH_ per well were cultured for 4 days in 200 μL R10 containing 2 μg/mL anti-CD28 (Tonbo 40–0289) and 10 U/mL IL-2 with an equal number of sorted CD8 T_FR_ (CD3+CD8+CXCR5^hi^CD44^hi^) or alone. At day 4, cells were stained with viability dye (Ghost Violet 450, Tonbo 13–0863) and analyzed by flow cytometry.

### Statistical analysis

Comparisons of uninfected and SIV-infected rhesus macaque spleen or lymph nodes were performed using non-parametric Mann Whitney tests. Comparisons of tonsil cultures were performed using unpaired Mann Whitney or Friedman non-parametric tests. In direct comparisons of paired data, a paired Wilcoxon ranked sums test was performed to compare the two group medians of interest. Significance is denoted in each figure by asterisks, as * = p < 0.05, ** = p< 0.01, and *** = p < 0.001. All statistical tests were performed with GraphPad Prism 6.

## Supporting Information

S1 Fig Human tonsil CD8 T_FR_ do not numerically increase after HIV infection. Tonsil cells were mock-spinoculated or spinoculated with X4- or R5-tropic HIV (n = 3, average shown). Cell numbers were determined using flow cytometry counting beads. Conventional CD8 T cells (blue line) and CD8 T_FR_ (red line) concentrations are shown for (A) mock-, (B) X4-, and (C) R5-spinoculated cells.
(TIF)

S2 Fig CD8 T_FR_ frequency and phenotype are not altered by cell culture conditions. Tonsil cells (n = 6) were stained immediately after isolation (Day 0) or mock-spinoculated and cultured for 2 days prior to flow staining (Day 2). Perforin and IL-10 expression levels were determined after 4 hours of PMA/ionomycin stimulation in the presence of GolgiPlug (brefeldin A). Statistical significance was determined by Wilcoxon matched-pairs tests.
(TIF)

S3 Fig A majority of CD8 T_FR_ are effector memory cells and are distinct from conventional CD8 T cells. Tonsil cells (n = 6) were isolated and immediately stained to determine effector (CD3+CD8+CD62L-CCR7-) and central (CD3+CD8+CD62L+CCR7+) memory populations. (A) PD-1 and CTLA-4 expression on effector and memory populations of CD8 T_FR_ and conventional CD8 T cells. (B) The frequency of effector and central memory cells in CD8 T_FR_ and conventional CD8 T cell populations and their expression of IL-10, CD122, GITR, and perforin. Perforin and IL-10 expression levels were determined after 4 hours of PMA/ionomycin stimulation in the presence of GolgiPlug (brefeldin A). Statistical significance was determined by Wilcoxon matched-pairs tests is displayed as * = p<0.05.
(JPG)

S4 Fig Human tonsil T_FH_ cytokine production is inhibited by CD8 T_FR_ but not CD8 conv. Tonsil cells were sorted into T_FH_, CD8 T_FR_, and CD8 conv populations. All cell populations were spinoculated with X4- or R5-tropic HIV and T_FH_ were cultured for 2 days alone or with CD8 T_FR_ or conventional CD8 T cells. (A) IL-2 production by T_FH_ co-cultured with increasing ratios of CD8 T_FR_ (n = 4). (B) IL-21 production (left) and IL-2 production (right) by T_FH_ co-cultured 1:1 with conventional CD8 T cells (n = 2). (C) Representative example of IL-21 production by T_FH_ co-cultured with conventional CD8 T cells and anti-Tim3 antibody (n = 6).
(TIF)

S5 Fig Proliferation of T_FH_ co-cultured with CD8 T_FR_. Tonsil cells were sorted into T_FH_, CD8 T_FR_, and CD8 conv populations. T_FH_ were stained with proliferation dye and cultured for 2 days either alone without stimulation (T_FH_, NS) or in the presence of stimulation anti-CD3/CD28 + IL-2 alone or 1:1 ratios with CD8 T_FR_ or CD8 conv (n = 5).
(TIF)

S6 Fig Tonsil conventional CD8 T cells do not suppress productive infection of T_FH_. Tonsil cells were sorted into T_FH_, CD8 T_FR_, and CD8 conv populations. Populations were spinoculated with X4- or R5-tropic GFP reporter HIV. T_FH_ were cultured for 2 days alone or 1:1 with CD8 T_FR_ or CD8 conv. Percent of GFP+ T_FH_ when cultured alone or with CD8 conv (X4: n = 7; R5: n = 6).
(TIF)

S7 Fig Correlations of rhesus macaque CD8 T_FR_ frequency to viral load. Correlation of CD8 T_FR_ frequencies in chronically SIV-infected rhesus macaques to plasma viral loads (n = 6). Statistical significance was determined by Spearman correlation tests.
(TIF)

S8 Fig Galectin-9 expression increases on CD8 T_FR_ in SIV-infected rhesus macaques. Disaggregated cells from lymph node and spleen of SIV-uninfected and SIV-infected rhesus macaques were analysed for galectin-9 expression on CD8 T_FR_ and CD8 conv (n = 6). Graphs depict median and range. Statistical significance was determined by one-way ANOVA and is displayed as * = p<0.05.
(TIF)

S9 Fig Conventional CD8 T cells from SIV-infected rhesus macaques do not suppress IL-21 via Tim-3 and do not induce apoptosis via HLA-E. Disaggregated cells from lymph node and spleen of SIV-infected rhesus macaques (n = 2) were sorted for T_FH_ and CD8 conv and co-cultured at a 1:1 ratio for 2 days with or without blocking antibody as noted and analysed by flow cytometry. (A) Representative flow plots showing percent T_FH_ producing IL-21, and (B) percent T_FH_ binding of Annexin-V.
(TIF)
