# Supplementary figures and images for: Follicular Regulatory CD8 T Cells Impair the Germinal Center Response in SIV and Ex Vivo HIV Infection
Source: PLoS Pathog. 2016 Oct 7;12(10):e1005924. doi: 10.1371/journal.ppat.1005924 (PMC5055335; doi:10.1371/journal.ppat.1005924)

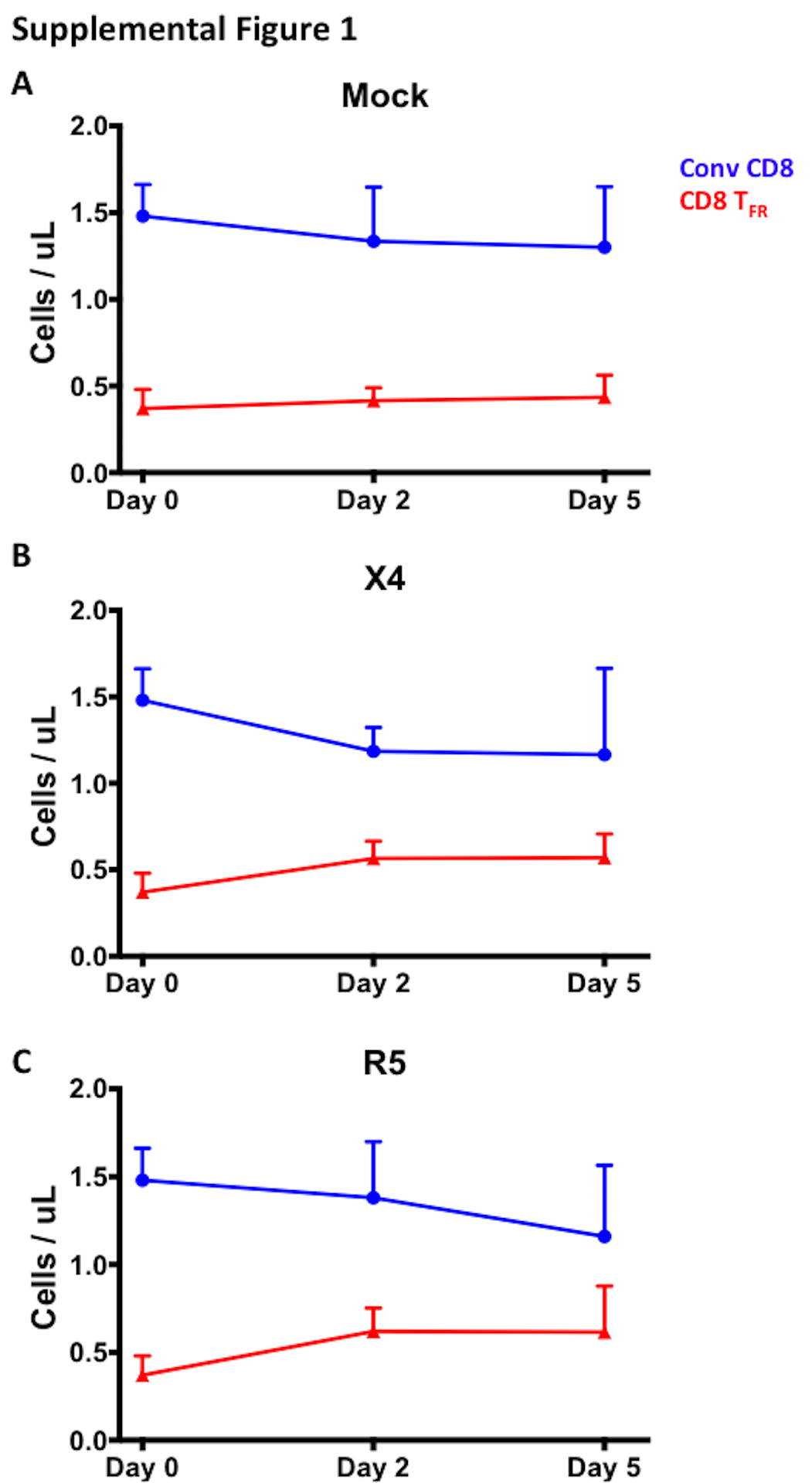

Supplement: S1 Fig — Tonsil cells were mock-spinoculated or spinoculated with X4- or R5-tropic HIV (n = 3, average shown). Cell numbers were determined using flow cytometry counting beads. Conventional CD8 T cells (blue line) and CD8 TFR (red line) concentrations are shown for (A) mock-, (B) X4-, and (C) R5-spinoculated cells. (TIF) [file ppat.1005924.s001.tif]

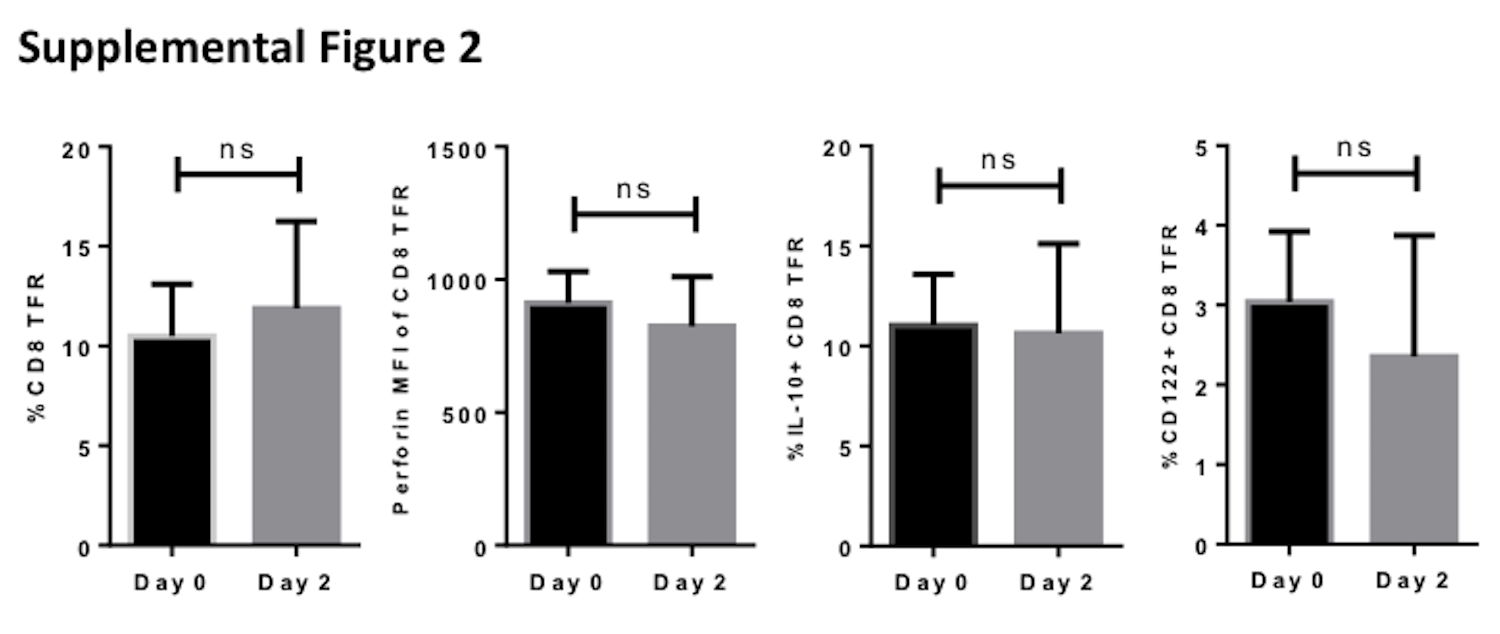

Supplement: S2 Fig — Tonsil cells (n = 6) were stained immediately after isolation (Day 0) or mock-spinoculated and cultured for 2 days prior to flow staining (Day 2). Perforin and IL-10 expression levels were determined after 4 hours of PMA/ionomycin stimulation in the presence of GolgiPlug (brefeldin A). Statistical significance was determined by Wilcoxon matched-pairs tests. (TIF) [file ppat.1005924.s002.tif]

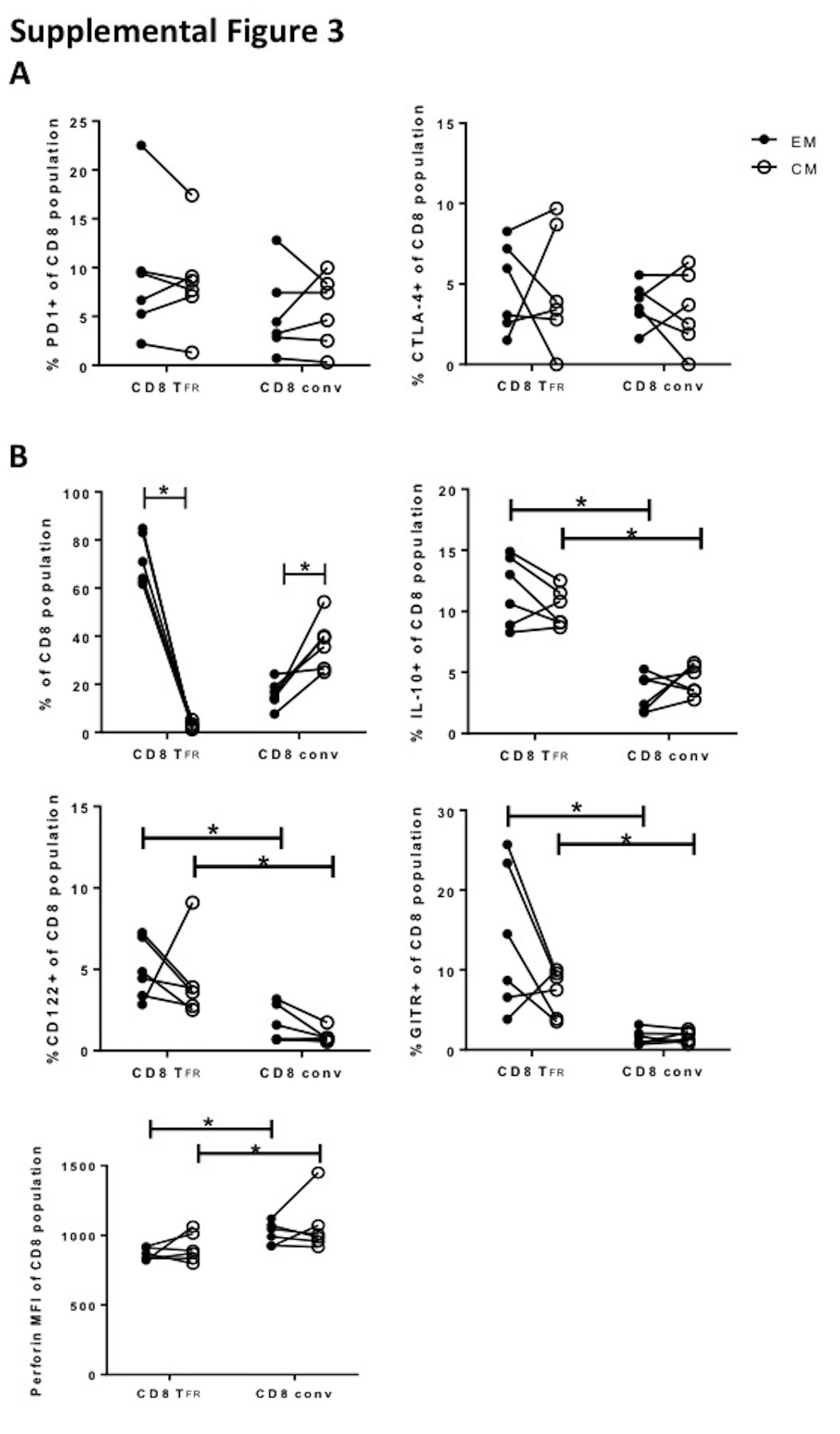

Supplement: S3 Fig — Tonsil cells (n = 6) were isolated and immediately stained to determine effector (CD3+CD8+CD62L-CCR7-) and central (CD3+CD8+CD62L+CCR7+) memory populations. (A) PD-1 and CTLA-4 expression on effector and memory populations of CD8 TFR and conventional CD8 T cells. (B) The frequency of effector and central memory cells in CD8 TFR and conventional CD8 T cell populations and their expression of IL-10, CD122, GITR, and perforin. Perforin and IL-10 expression levels were determined after 4 hours of PMA/ionomycin stimulation in the presence of GolgiPlug (brefeldin A). Statistical significance was determined by Wilcoxon matched-pairs tests is displayed as * = p<0.05. (JPG) [file ppat.1005924.s003.jpg]

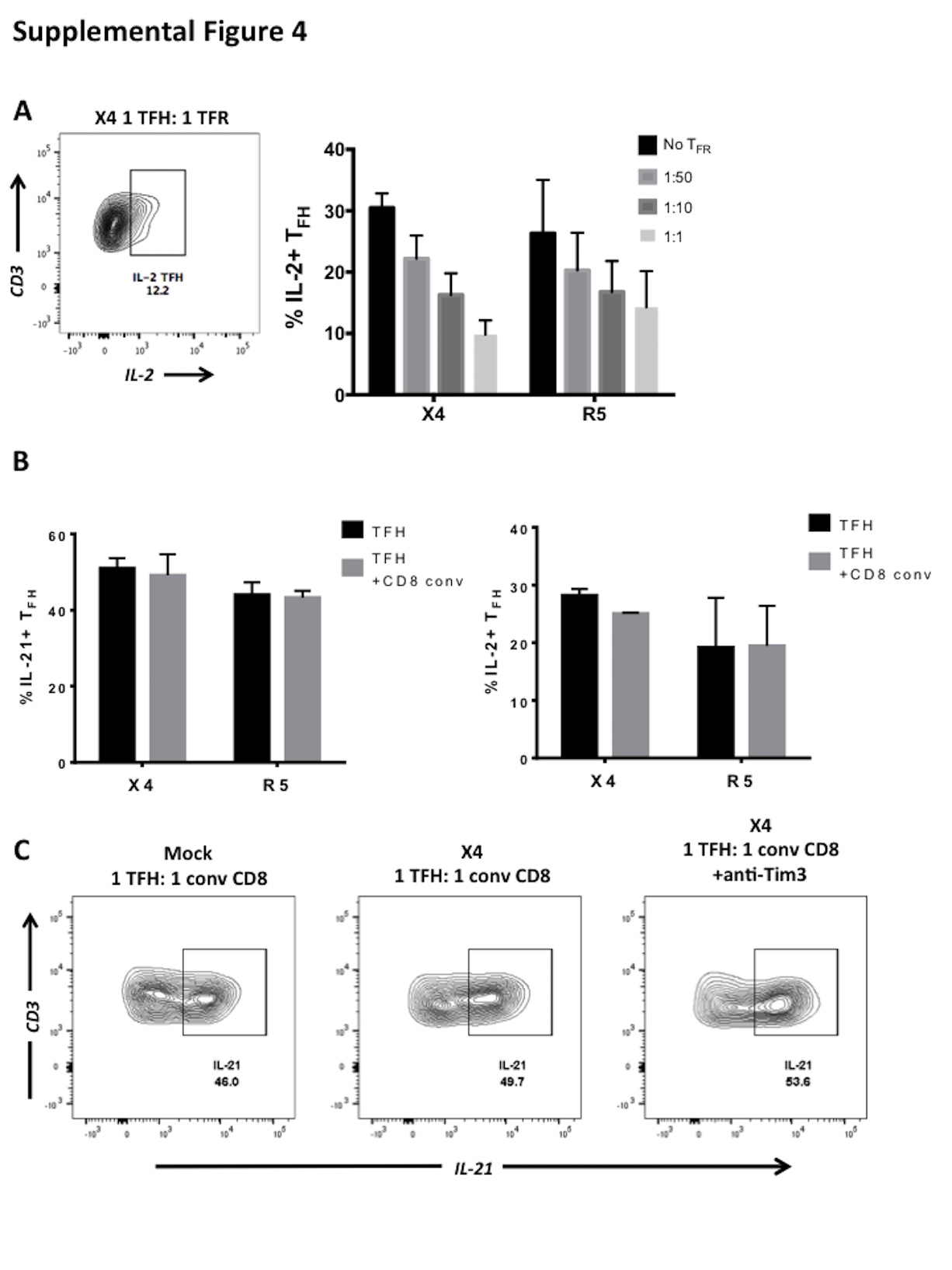

Supplement: S4 Fig — Tonsil cells were sorted into TFH, CD8 TFR, and CD8 conv populations. All cell populations were spinoculated with X4- or R5-tropic HIV and TFH were cultured for 2 days alone or with CD8 TFR or conventional CD8 T cells. (A) IL-2 production by TFH co-cultured with increasing ratios of CD8 TFR (n = 4). (B) IL-21 production (left) and IL-2 production (right) by TFH co-cultured 1:1 with conventional CD8 T cells (n = 2). (C) Representative example of IL-21 production by TFH co-cultured with conventional CD8 T cells and anti-Tim3 antibody (n = 6). (TIF) [file ppat.1005924.s004.tif]

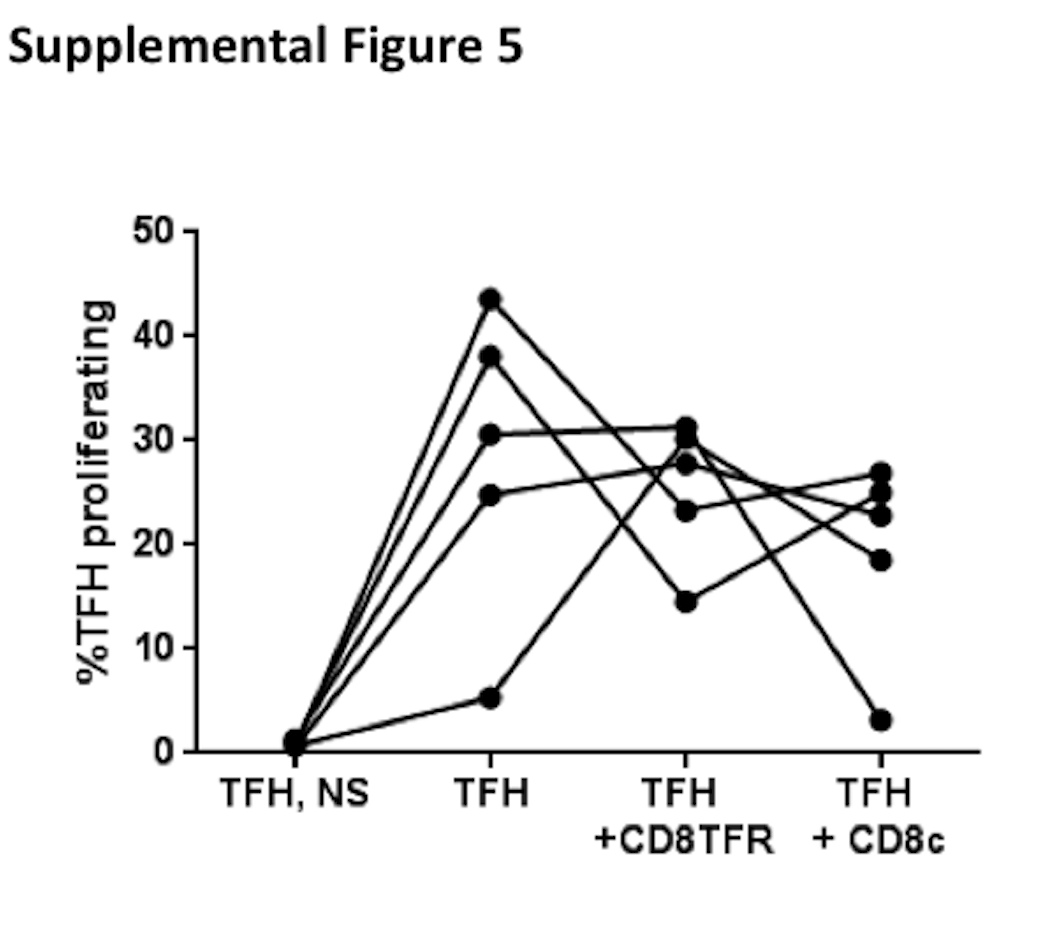

Supplement: S5 Fig — Tonsil cells were sorted into TFH, CD8 TFR, and CD8 conv populations. TFH were stained with proliferation dye and cultured for 2 days either alone without stimulation (TFH, NS) or in the presence of stimulation anti-CD3/CD28 + IL-2 alone or 1:1 ratios with CD8 TFR or CD8 conv (n = 5). (TIF) [file ppat.1005924.s005.tif]

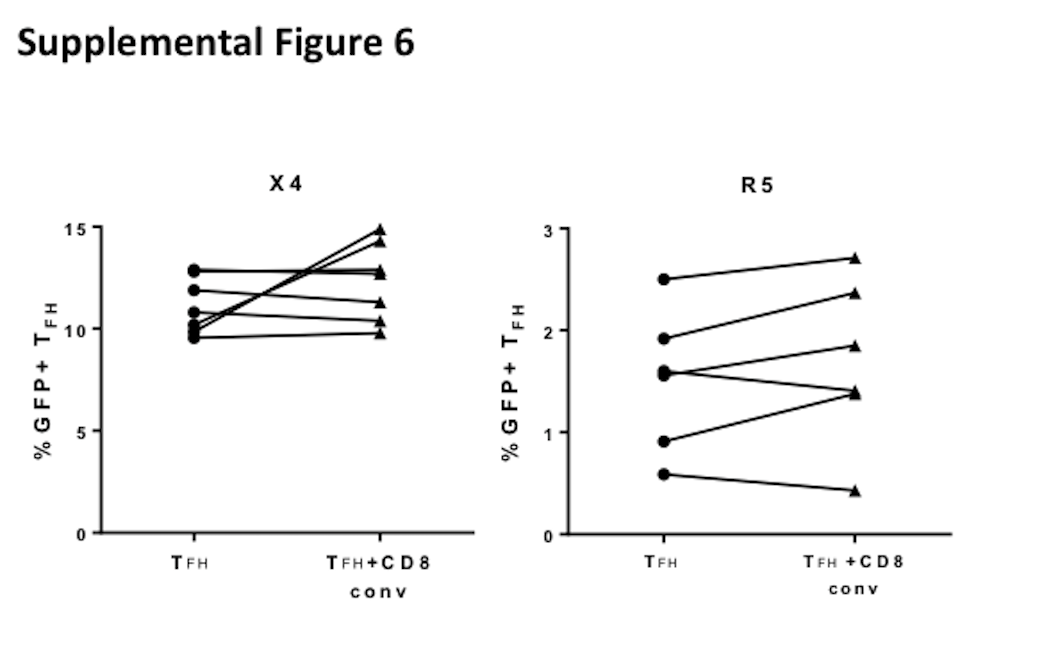

Supplement: S6 Fig — Tonsil cells were sorted into TFH, CD8 TFR, and CD8 conv populations. Populations were spinoculated with X4- or R5-tropic GFP reporter HIV. TFH were cultured for 2 days alone or 1:1 with CD8 TFR or CD8 conv. Percent of GFP+ TFH when cultured alone or with CD8 conv (X4: n = 7; R5: n = 6). (TIF) [file ppat.1005924.s006.tif]

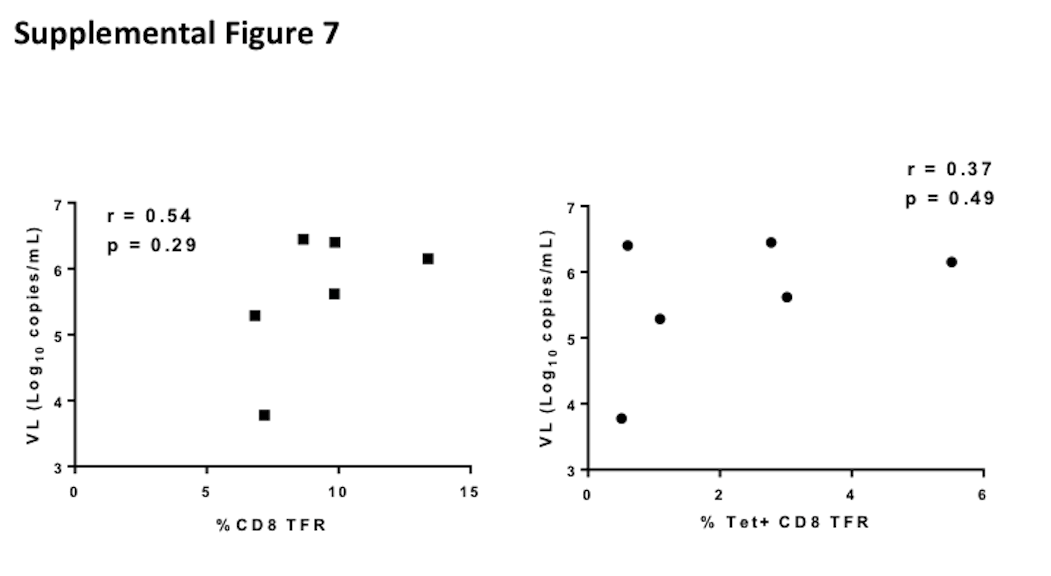

Supplement: S7 Fig — Correlation of CD8 TFR frequencies in chronically SIV-infected rhesus macaques to plasma viral loads (n = 6). Statistical significance was determined by Spearman correlation tests. (TIF) [file ppat.1005924.s007.tif]

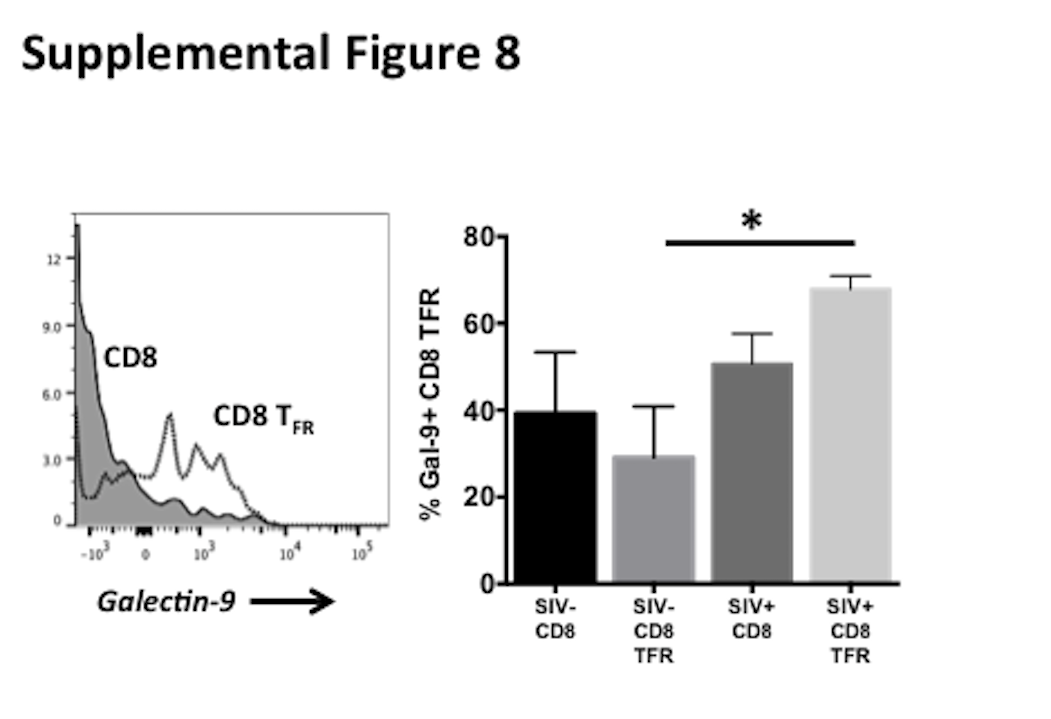

Supplement: S8 Fig — Disaggregated cells from lymph node and spleen of SIV-uninfected and SIV-infected rhesus macaques were analysed for galectin-9 expression on CD8 TFR and CD8 conv (n = 6). Graphs depict median and range. Statistical significance was determined by one-way ANOVA and is displayed as * = p<0.05. (TIF) [file ppat.1005924.s008.tif]

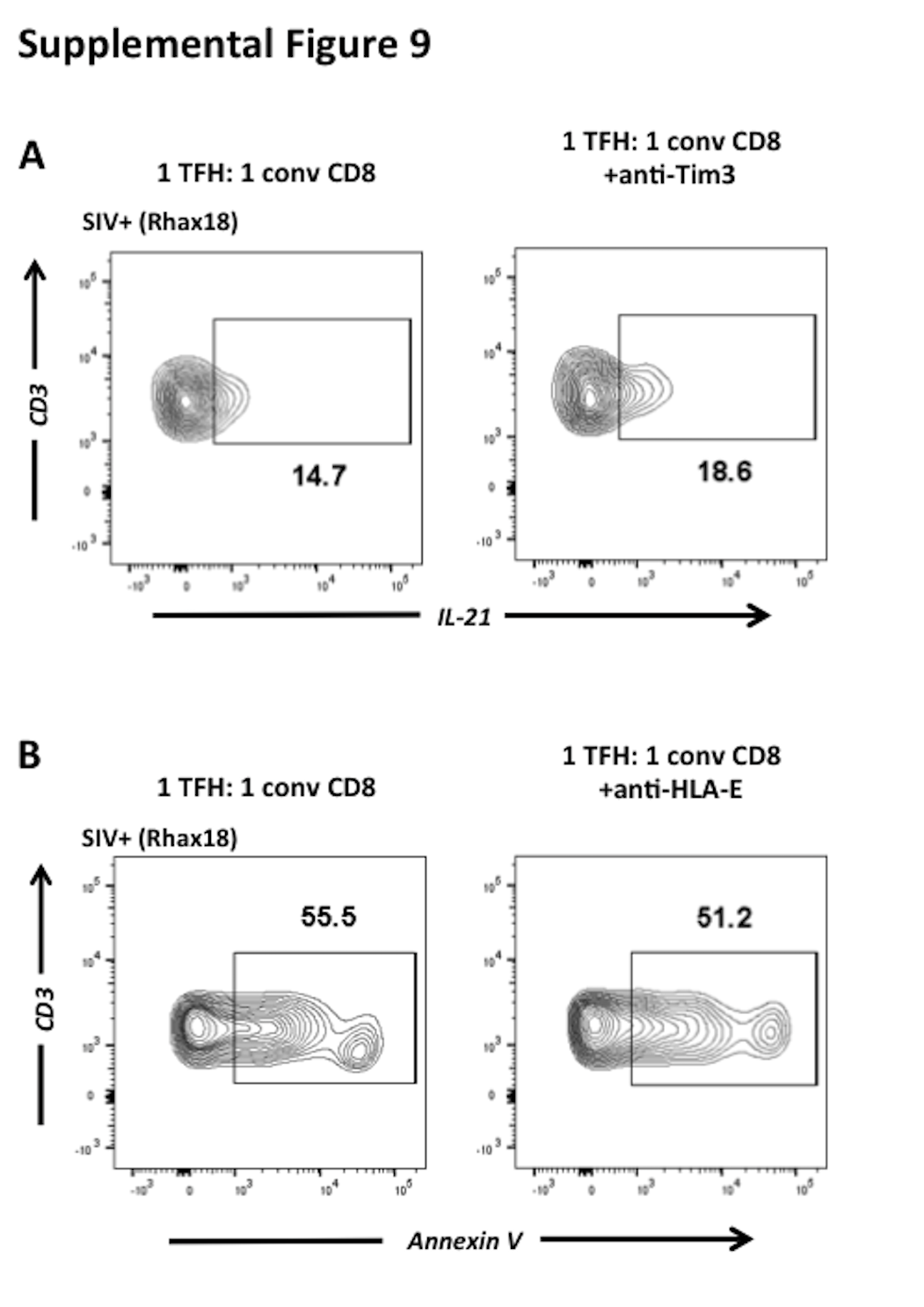

Supplement: S9 Fig — Disaggregated cells from lymph node and spleen of SIV-infected rhesus macaques (n = 2) were sorted for TFH and CD8 conv and co-cultured at a 1:1 ratio for 2 days with or without blocking antibody as noted and analysed by flow cytometry. (A) Representative flow plots showing percent TFH producing IL-21, and (B) percent TFH binding of Annexin-V. (TIF) [file ppat.1005924.s009.tif]
